# Chemical Elicitors Induce Rare Bioactive Secondary Metabolites in Deep-Sea Bacteria under Laboratory Conditions

**DOI:** 10.3390/metabo11020107

**Published:** 2021-02-12

**Authors:** Rafael de Felício, Patricia Ballone, Cristina Freitas Bazzano, Luiz F. G. Alves, Renata Sigrist, Gina Polo Infante, Henrique Niero, Fernanda Rodrigues-Costa, Arthur Zanetti Nunes Fernandes, Luciane A. C. Tonon, Luciana S. Paradela, Renna Karoline Eloi Costa, Sandra Martha Gomes Dias, Andréa Dessen, Guilherme P. Telles, Marcus Adonai Castro da Silva, Andre Oliveira de Souza Lima, Daniela Barretto Barbosa Trivella

**Affiliations:** 1 Brazilian Biosciences National Laboratory (LNBio), National Center for Research in Energy and Materials (CNPEM), Campinas 13083-970, SP, Brazil; rafael.felicio@lnbio.cnpem.br (R.d.F.); patyballone@gmail.com (P.B.); cristina.bazzano@lnbio.cnpem.br (C.F.B.); luiz.alves@lnbio.cnpem.br (L.F.G.A.); renata.sigrist@lnbio.cnpem.br (R.S.); imaginapolo@gmail.com (G.P.I.); henrique.niero@lnbio.cnpem.br (H.N.); fernanda-frc@hotmail.com (F.R.-C.); zanetti.arthur@gmail.com (A.Z.N.F.); luciane.chimetto@gmail.com (L.A.C.T.); lucianasparadela@gmail.com (L.S.P.); rennakaroline@gmail.com (R.K.E.C.); sandra.dias@lnbio.cnpem.br (S.M.G.D.); andrea.dessen@lnbio.cnpem.br (A.D.); 2 Institute of Biology, University of Campinas (UNICAMP), Campinas 13083-862, SP, Brazil; 3 Institute of Computing (IC), University of Campinas (UNICAMP), Campinas 13083-852, SP, Brazil; gpt@ic.unicamp.br; 4 Institut de Biologie Structurale (IBS), Université Grenoble Alpes, CNRS, CEA, F-38000 Grenoble, France; 5 School of Sea, Science and Technology, University of Vale do Itajaí (Univali), Itajaí 88302-202, SC, Brazil; marcus.silva@univali.br (M.A.C.d.S.); lima@univali.br (A.O.d.S.L.)

**Keywords:** bacterial natural products, natural product libraries, drug discovery, chemical elicitors, cryptic gene clusters, chemical space, molecular networking, dereplication, deep-sea bacteria, LC-MS/MS data mining

## Abstract

Bacterial genome sequencing has revealed a vast number of novel biosynthetic gene clusters (BGC) with potential to produce bioactive natural products. However, the biosynthesis of secondary metabolites by bacteria is often silenced under laboratory conditions, limiting the controlled expression of natural products. Here we describe an integrated methodology for the construction and screening of an elicited and pre-fractionated library of marine bacteria. In this pilot study, chemical elicitors were evaluated to mimic the natural environment and to induce the expression of cryptic BGCs in deep-sea bacteria. By integrating high-resolution untargeted metabolomics with cheminformatics analyses, it was possible to visualize, mine, identify and map the chemical and biological space of the elicited bacterial metabolites. The results show that elicited bacterial metabolites correspond to ~45% of the compounds produced under laboratory conditions. In addition, the elicited chemical space is novel (~70% of the elicited compounds) or concentrated in the chemical space of drugs. Fractionation of the crude extracts further evidenced minor compounds (~90% of the collection) and the detection of biological activity. This pilot work pinpoints strategies for constructing and evaluating chemically diverse bacterial natural product libraries towards the identification of novel bacterial metabolites in natural product-based drug discovery pipelines.

## 1. Introduction

Secondary metabolites, also known as natural products, are complex and three-dimensionally oriented molecules that belong to diverse groups of organic compounds, which expand across the chemical space of known molecules [1]. Notably, secondary metabolites are important sources of chemical diversity for drug discovery and development [2].

Ecologically, secondary metabolites are involved in the adaptive processes of species, interacting effectively and specifically with their biological targets [1,2]. Microorganisms, especially bacteria and fungi, are sources of a vast chemical diversity of metabolites, due to their constant interaction with widely colonized areas, which are competitive habitats under constant stress [3]. To increase survival rates under these conditions, microorganisms adopt several strategies, such as the production of secondary metabolites to counteract physicochemical changes in the environment (e.g., pigments), as well as bioactive secondary metabolites to combat their competitors in their native habitat. Examples of such metabolites are penicillin and epoxyketone peptides. The first, produced by fungi of the genera *Penicillium*, is induced in the presence of a bacteria, combating these competitors by covalently inhibiting Penicillin-Binding Proteins (PBPs), many of which are essential for bacterial cell-wall synthesis and stability [4]. Beta-lactam antibiotics, such as penicillin, are one of the main classes of antibacterial agents used by humanity. On the other hand, the epoxyketone peptides eponemycin [5] and epoxomicin [6] are produced by actinomycete bacteria, selectively binding to the active sites of the proteasome, an important proteolytic protein complex of eukaryotic cells, essential for protein homeostasis [7,8]. Along with their antifungal properties, proteasome inhibitors of bacterial origin have been crucial for inspiring the development of second (e.g., carfilzomib/Kyprolis^®^—derived from epoxomicin [6]) and third (e.g., salinosporamide A/Marizomib [9]) generation proteasome inhibitors, with wide anticancer applications [10].

However, finding and producing bioactive secondary metabolites under controlled artificial conditions is not an easy task. The biosynthesis of secondary metabolites by bacteria is a well-regulated process, involving groups of genes often organized into biosynthetic gene clusters (BGCs) within the bacterial genome. Each BGC can code for numerous biosynthetic enzymes, regulators and sometimes macromolecules that confer resistance to the final natural product [11,12]. The regulation of BGC expression provides bacteria with a way to expend energy on secondary metabolite production only when it is essential for growth or ecological competition [13]. BGC expression might be regulated by a variety of molecular mechanisms, and thus BGC regulation represents a hurdle for obtaining natural products under laboratory conditions, as most of cultivable bacteria do not express their biosynthetic potential outside of their native environment [14]. Therefore, the need to implement alternative and innovative methodologies to induce the expression of silent and/or poorly expressed BGCs to produce novel bioactive natural substances under controlled conditions is evident [15].

Bacterial elicitation is a strategy being pursued by the natural product community [14,15,16,17]. It has been shown that physical and chemical alterations of growth media may alter the transcription rate of certain genes and may result in the induction of BGC expression otherwise kept silent under laboratory culture conditions. These alterations can involve the composition of growth media or growth conditions (e.g., pH, temperature, oxygenation), co-cultivation with competitors, addition of precursors or the use of chemicals that act as specific or broad epigenetic elicitors [17]. However, to the best of our knowledge, a systematic evaluation of such methods and the correlation between the growth condition and the elicitation method, the secondary metabolites produced and biological relevance of the produced compounds, is absent from the literature.

Metabolic changes provoked by chemical elicitors can be monitored by hyphenated liquid chromatography-tandem mass spectrometry (LC-MS/MS), revealing how elicitors can globally modify the expression of bacterial natural products, even at trace levels. However, LC-MS/MS data processing and mining remain challenging due to the inherent properties of metabolite elution in the LC and its behavior in electrospray ionization (ESI). These challenges have been tackled by the scientific community, including our group, initially focusing on the subsequent experimental problems that can be handled by computational methods, using mathematical rules and the physicochemical logic behind the data. (i) LC provides the separation of stereoisomers, that may display very similar MS/MS spectra. The retention time of the MS/MS must be taken into account, thereby avoiding merging isomeric MS/MS spectral pairs during LC-MS/MS data processing. In addition, the peak area of elution related to a given single compound is important information for the relative quantification of compounds in the sample, and may also be annotated and used during LC-MS/MS data mining. (ii) Variations in ion formation by a given compound during ESI are often observed. For instance, the formation of adducts, dimers, multiple charges and in-source fragmentation are frequently observed leading to MS/MS data amplification (about 40–80% of the collected MS/MS spectra [18]) and loss of information of the MS/MS spectra related to the original compound. If not treated correctly, dataset dereplication with MS/MS databases of known compounds is further compromised, since spectral identification can be misled by spectral similarities to ionization variants instead of to the real metabolite. In addition to proprietary and publicly available LC-MS/MS data processing and mining tools, many advances in LC-MS/MS data handling have been recently reported, such as TidyMS for data extraction [19], mzAdan [20] and feature-based molecular networking [21] to account for ionization variants, and CANOPUS for systematic compound class annotation based on MS/MS data [22]. While these works were being reported, we developed our own tool, the NP^3^ MS workflow, for processing and mining of LC-MS/MS data obtained from unpurified natural product chemical libraries.

In this work, we report a pilot library—the elicited pre-fractionated bacterial (EPfB) chemical library—which was prepared using a new methodology to select chemical elicitors aiming to modulate deep-sea bacterial metabolism. The investigation of the chemical diversity of the EPfB library was performed by frontier cheminformatic methods using MS/MS spectra, compounds and networks derived from the NP^3^ MS workflow data processing, assisted by the Global Natural Product Social Molecular Networking (GNPS) [23] and the Universal Natural Products Database (UNPD)—In-Silico MS/MS DataBase (ISDB) [24] databases for compound identification. The biological potential of the EPfB library was assessed by comparing the chemical space achieved by the library with that of drugs and natural products, and further by screening the library in four bioassays. We show that the EPfB library’s components overlap with the chemical space of drugs. Furthermore, chemical elicitation promoted the production of rare or potentially novel natural products, and bioactivity was mainly detected in the elicited pre-fractionated samples. These findings corroborate the statement that chemical elicitation and natural product library pre-fractionation are key strategies that allow the exploration of unique bioactive bacterial secondary metabolites. This work further pinpoints strategies for constructing and mining chemically diverse bacterial natural product libraries for natural product-based drug discovery pipelines.

## 2. Results

**Figure 1:**
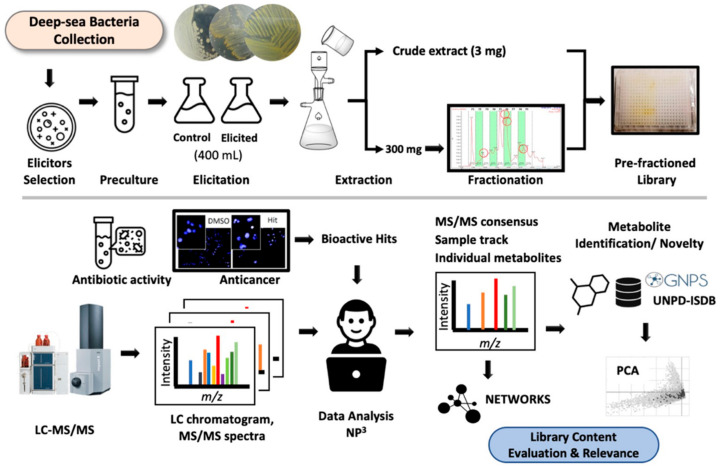
Workflow for the construction (upper panel) and analysis (bottom panel) of the elicited pre-fractionated bacterial EPfB chemical library discussed in the present work.

The pilot EPfB chemical library was prepared following the workflow presented in the upper panel of Figure 1. This workflow was directed towards the induction of cryptic BGCs in deep-sea bacteria under laboratory conditions, aiming to appraise the vast chemical diversity that can be provided by a bacterial collection. EPfB library’s content and relevance were then assessed (Figure 1, bottom panel) using state-of-the-art cheminformatic methods and biological assays.

The steps for the construction of the EPfB library started from the test and selection of chemical elicitors and culture conditions, using the new elicitation test reported in Section 2.1.2. Once selected, bacteria were grown in the presence or absence of the chemical elicitors using large-scale cultivation (Section 2.1.3). Metabolites were extracted and fractionated by preparative HPLC. The EPfB library was assembled in screening plates, containing 18 crude extracts and 162 enriched fractions (total = 180 samples), aiming the evaluation of chemical elicitation and library pre-fractionation on the production and detection of novel or bioactive bacterial metabolites.

The entire EPfB chemical library was evaluated in four biological assays, returning four fractions confirmed as bioactive in these assays (Section 2.2.4).

For investigation of the library’s chemical composition, high-resolution LC-MS/MS data were acquired from all the samples of the EPfB library. LC-MS/MS data were processed with the NP^3^ MS workflow, which returned the [M + H]^+^ spectra relative to bacterial metabolites detected from individual EPfB library’s samples (please see Methods for details). The [M + H]^+^ spectra were further dereplicated against available spectra databases of known natural products (GNPS and UNPD-ISDB), returning possible chemical structures representing the bacterial metabolites contained in the EPfB library.

The collection of annotated [M + H]^+^ spectra was counted according to their detection in each sample group (control vs. elicited vs. both; crude extract vs. enriched fractions), and further for their identification against spectra databases (identified vs. non-identified spectra). This was done aiming to numerically dissect the overall contribution of bacterial elicitation and crude extract fractionation on the production and detection of bacterial metabolites, respectively, and further on the novelty of the library (Section 2.2.1).

The annotated [M + H]^+^ spectra collection was further visualized using spectra similarity molecular networking (SSMN) and principal component analysis (PCA). SSMN was used to organize the EPfB spectra in terms of similarity, allowing for the visualization of clusters of similar metabolites (possible analogues or compound families) and further to overview the distribution of the elicited metabolites across the similarity clusters (Section 2.2.3). The distribution of the identified metabolites could also be visualized in the SSMN (represented as squares in Section 2.2.3).

PCA was used with four main objectives: (i) to compare the chemical space of identified compounds of the EPfB library with known natural products and approved drugs (Section 2.2.4); (ii) to map the contribution of non-identified analogues (non-identified spectra, however directly connected to an identified node in the SSMN—Section 2.2.5); (iii) for dissecting the influence of chemical elicitors on the chemical space covered by the EPfB library (Section 2.2.5); and (iv) the contribution of minor compounds (detected only in enriched fractions) to the chemical space covered by the EPfB library (Section 2.2.5).

Additionally, we used the established workflow for analyzing the contribution of each strain to the EPfB library, and the influence of chemical elicitors in individual strains. These analyses are presented as Supplementary Materials.

### 2.1. Construction of the Elicited Pre-Fractionated Bacterial (EPfB) Chemical Library

#### 2.1.1. Collection of Deep-Sea Bacteria

For the construction of the EPfB chemical library, bacterial strains were selected from a collection of isolated deep-sea marine microorganisms, the LAMA collection, maintained at the University of Itajai Valley (Univali-SC) and curated by Professors André Oliveira de Souza Lima and Marcus Adonai Castro da Silva. This collection is potentially one of the major sources of substances that are still under-explored in the context of the discovery of bioactive natural compounds. Nineteen strains from the LAMA collection were chosen for the purposes of this pilot study. These strains belong to the Actinobacteria, Firmicutes and Proteobacteria phyla.

#### 2.1.2. Implementation of a New Method for Testing Growth Conditions and Chemical Elicitors for Bacterial Natural Product Library Construction

Determination of the best growth condition is essential for inducing the production of chemically diverse metabolites by a given bacteria. Therefore, optimal culture conditions were selected based on bacterial population density for each strain and the tested chemical elicitors. The tests were performed with eight reported chemical elicitors: procaine and sodium butyrate, known for their epigenetic action in bacteria by inhibiting DNA-methyltransferases [25] and histone deacetylase (HDAC) [17], respectively; ampicillin, kanamycin, chloramphenicol, and streptomycin sulfate, which are antibiotics and were selected aiming to mimic the presence of natural competitors; dimethyl sulfoxide (DMSO), a known elicitor of a variety of bacterial metabolites with effects at the transcriptional level [14]; and ethylenediaminetetraacetic acid (EDTA), a metal chelator used with the intention to induce the production of bacterial siderophores or other metabolites. The effect of elicitor concentration was also evaluated, resulting in a total of 17 different elicited conditions tested for each strain (8 chemical elicitors tested in at least 2 concentrations, streptomycin sulfate being tested at 3 concentrations due to positive previous results in our group at the selected concentrations).

**Figure 2:**
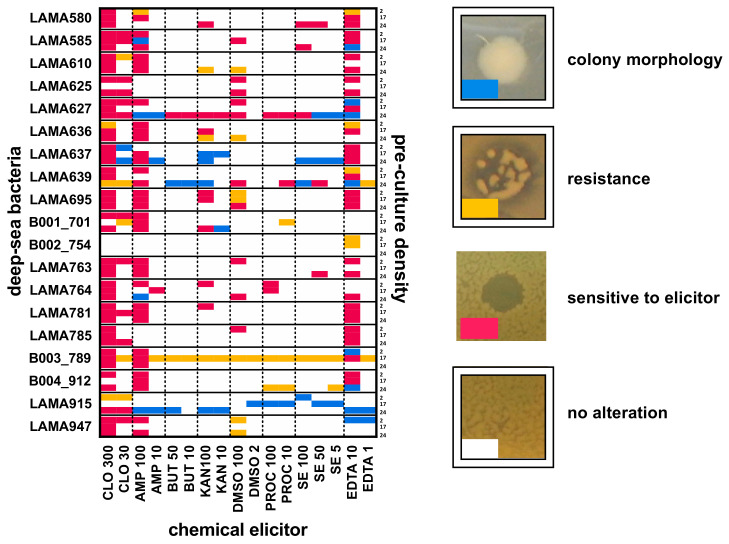
Testing bacterial growth conditions and chemical elicitors in solid media reveals alterations in colony morphology, indicating the modulation of bacterial metabolism. Graphical representation of morphological changes in bacterial growth in solid media induced by the different chemical elicitors, in different growth conditions. Each row represents a bacterial strain at a given preculture density (2, 17 or 24 h—strain sub-rows). Each column represents a chemical elicitor. The classification scheme adopted for annotating the morphological changes produced by chemical elicitors is represented by different colors: no changes (white); halo formation, sensitive to elicitor (magenta); bacterial growth inside the halo, microbial resistance (yellow); changes in colony morphology (blue). Chemical elicitors: CLO 300: chloramphenicol 300 µg/mL; CLO 30: chloramphenicol 30 µg/mL; AMP 100: ampicillin 100 µg/mL; AMP 10: ampicillin 10 µg/mL; BUT 50: 50 µM sodium butyrate; BUT10: 10 mM sodium butyrate; KAN 100: kanamycin 100 µg/mL; KAN 10: kanamycin 10 µg/mL; DMSO 100: 100% DMSO; DMSO 2: 2% DMSO; PROC100: 100 µM procaine; PROC 10: 10 µM procaine; SE 5: streptomycin sulfate 5 µg/mL; SE 50: streptomycin sulfate 50 µg/mL; SE100: streptomycin sulfate 100 µg/mL; EDTA 10: 10 mM EDTA; EDTA 1: 1 mM EDTA.

The preliminary tests were evaluated according to visible morphological changes caused by the elicitor addition to solid media in the presence of a bacterial film. The classification of these alterations was qualitative, performed by evaluating the morphology of colonies and classifying each elicitor according to the alterations it caused. Furthermore, each of the 19 bacterial strains was evaluated at three different population densities (2, 17 and 24 h of preculture, optic densities at 600 nm varying from OD600 0.2 to OD600 5.0 a.u.), accounting for 969 tested cultivation conditions (19 strains, 17 elicited conditions and 3 pre-culture densities). For a better presentation of the results, the data were plotted in a matrix representing the observed morphological changes in different colors (Figure 2).

In this matrix, it is clear that some bacterial strains (rows) presented high susceptibility to morphological changes with different chemical elicitors and concentrations (columns). The antibiotics chloramphenicol and ampicillin were lethal for most bacterial strains at the highest concentration. However, at sublethal concentrations, resistance (e.g., LAMA610, LAMA639, B001_701, B003_789) or changes in colony morphology (e.g., LAMA627, LAMA637) were observed, potentially indicating the modulation of bacterial metabolism. Some strains, such as LAMA627, LAMA639, B003_789 and LAMA915, were highly susceptible to the tested elicitors, presenting morphological changes in almost all the tests performed. The population density also influenced the morphological changes, as can be observed for the strain LAMA915, that showed distinct responses to different elicitors depending on the populational density, more significantly at the highest densities (17 h and 24 h of pre-cultures).

The tests on solid media were designed to be an easy and low-cost method for initial investigation of the culture condition, elicitor effect on bacterial response and, possibly, on secondary metabolite production. Once validated, this test can be easily implemented, guiding the selection of bacterial strains and elicitation conditions for preparing bacterial natural product libraries.

#### 2.1.3. Preparation of the EPfB Chemical Library

Based on the results presented in Figure 2, LAMA627 (ampicillin 100 µg/mL), LAMA639 (kanamycin 100 µg/mL), B002_754 (EDTA 10 mM), B003_789 (EDTA 10 mM), B004_912 (EDTA 10 mM), LAMA915 (procaine 100 µM; ampicillin 100 µg/mL and EDTA 10 mM) and LAMA585 (sodium butyrate 50 µM; ampicillin 100 µg/mL and EDTA 10 mM) were selected for large scale cultivation (400 mL), along with a control growth condition for each strain prepared in the absence of elicitors (controls). Bacterial crude extracts were fractionated by reverse-phase HPLC, rendering 9 fractions each. Crude extracts and fractions were assembled into a pilot screening library composed of 180 samples (18 crude extracts and 162 enriched fractions), plus 18 culture media.

The EPfB library was then analyzed by LC-MS/MS for evaluation of its chemical composition. The library was further tested in four bioassays: proteasome inhibition, triple-negative breast cancer cell-based assay targeted for glutaminase inhibition, and Gram-positive and Gram-negative bacterial growth inhibition.

### 2.2. Chemical Composition of the EPfB Chemical Library

LC-MS/MS data from all samples were jointly processed using the NP^3^ MS workflow (vide Methods), aiming to determine the chemical composition of the collection, as well as the contribution of chemical elicitation and crude extract fractionation to the detected chemical diversity of the EPfB library. For this, the MS/MS consensus spectra relative to the putative [M + H]^+^ ions were extracted and globally analyzed using molecular networking. These spectra were further compared to external MS/MS databases for initial metabolite identification, and unmatched spectra were associated to the novelty of the chemical collection. The identified compounds were additionally used for mapping the chemical diversity and the biological relevance of the EPfB library.

#### 2.2.1. Global Analysis of the Bacterial Metabolites Contained in the EPfB Library

**Figure 3:**
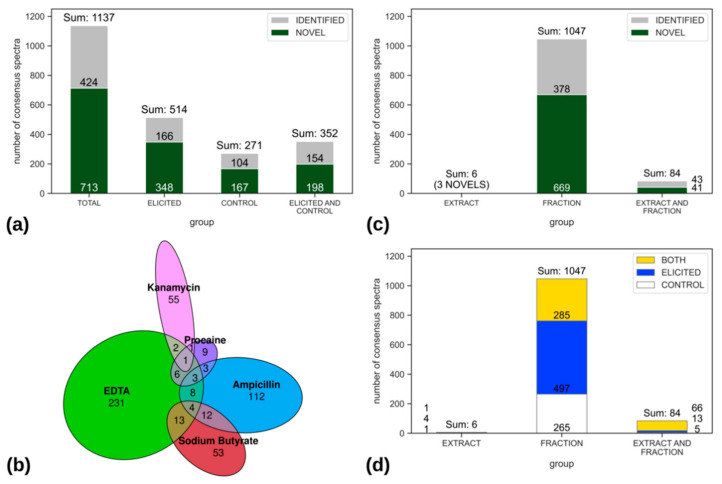
MS/MS consensus spectra distribution across the working groups reveals that the elicitation and fractionation strategies used for the bacterial chemical library construction increased the number and novelty of the metabolites detected by LC-MS/MS. (**a**) Distribution of total MS/MS consensus spectra related to [M + H]+ ions linked to bacterial metabolites (spectra related to chromatographic blanks, culture media and elicitors were excluded), in the different bacterial growth conditions: exclusively in the elicited conditions (elicited), non-elicited conditions (control) and in both elicited and control conditions. Bars are colored according to matches with databases (match = identified (grey), unmatched = potentially novel compound (green)). The cut-off for considering a spectrum as identified was MQScore ≥ 0.2, for the UNPD-ISDB (in silico), and MQScore ≥ 0.65, for GNPS (experimental) databases, with the MQScore ranging from 0 (totally dissimilar) to 1 (completely identical). (**b**) Euler diagram distribution of themetabolites detected exclusively under the elicited conditions: EDTA 10 mM, ampicillin 100 µg/mL, sodium butyrate 50 µM, kanamycin 100 µg/mL and procaine 100 µM. (**c**) Distribution of the metabolites detected exclusively in crude extracts (extract), fractions and both extract and fraction samples. Bars are color coded according to compound identification in databases—the same criteria used in (**a**) was applied. (**d**) Distribution of the metabolites detected in crude extracts, fractions and both extract and fraction samples, colored according to their detection in control (white), elicited (blue) or both (yellow—control and elicited) growth conditions.

The LC-MS/MS dataset processed from the 180 library samples resulted in the detection of 1137 MS/MS [M + H]^+^ consensus spectra linked to unique bacterial metabolites (Figure 3a). The elicited conditions contributed to 45% (514/1137) of these metabolites, which were exclusively detected in samples from bacterial cultures grown in the presence of the chemical elicitors and would not be observed under typical growth conditions. At the same time, 24% (271/1137) of the bacterial metabolites were only found in the control exclusive conditions, being thus downregulated in the presence of chemical elicitors. Finally, about 31% (352/1137) of the metabolites were detected in both control and elicited samples of the EPfB library, thus not being influenced by chemical elicitors.

EDTA and ampicillin were used for eliciting 5 and 3 strains respectively, being the chemical elicitors that most contributed to the total number of elicited metabolites, each representing an average of 50 new metabolites per strain (Figure 3b). Kanamycin, procaine and sodium butyrate were used in individual strains, further adding elicited metabolites to the collection. Sodium butyrate contributed with the highest number of metabolites per strain, eliciting 82 and adding 53 new compounds to the elicited group.

Next, we analyzed the impact of crude extract fractionation on the detection of bacterial metabolites by LC-MS/MS. Most of the detected compounds were found exclusively in fractions (1047/1137—92%), being those missed in the crude extracts (Figure 3c). On the other hand, very few (*n* = 6) compounds were missed by fractionating the crude extracts. In addition, a comparative analysis of crude extracts and fractions revealed that potentially novel compounds (669/713—94%) were exclusively found in the fractions (Figure 3c). Furthermore, ~97% of the compounds were detected exclusively in the elicited (497/514) or control groups (265/271), and 81% (285/352) of the common compounds (found in both, control and elicited growth conditions) were only found in the fractions (Figure 3d). Metabolites detected exclusively in the fractionated samples probably represent minor or low ionization energy molecules, for which the detection could be suppressed during LC-MS/MS data acquisition from highly complex samples, such as crude extracts. These minor compounds were evidenced in the enriched fractions and were modulated by chemical elicitation, as they were mostly found in the elicited or control exclusive fractions.

#### 2.2.2. Compound Novelty Introduced by Eliciting Bacterial Cultures

The [M + H]^+^ consensus spectra retrieved with the NP^3^ MS workflow data process were compared to GNPS [23] and to UNPD-ISDB [24] databases. From the 1137 spectra related to bacterial metabolites, 424 (37%) matched with MS/MS spectra of known natural products. Considering that UNPD-ISDB alone contains 213,210 out of the ~300,000 natural products already reported, it is possible that the remaining 713 (63%) non-identified spectra found in the EPfB library represent novel bacterial metabolites (Figure 3a).

The elicited conditions added 49% (348/713) of the novel MS/MS spectra. In terms of relative novelty, 68% (348/514) of the spectra found exclusively in the elicited conditions are unmatched with databases versus 62% (167/271) of the spectra exclusive to control, and 56% (198/352) of the common spectra (found in both control and elicited conditions)—Figure 3a, showing that chemical elicitors could mostly modulate the expression of novel bacterial metabolites.

#### 2.2.3. Chemical Diversity of the EPfB Library

**Figure 4:**
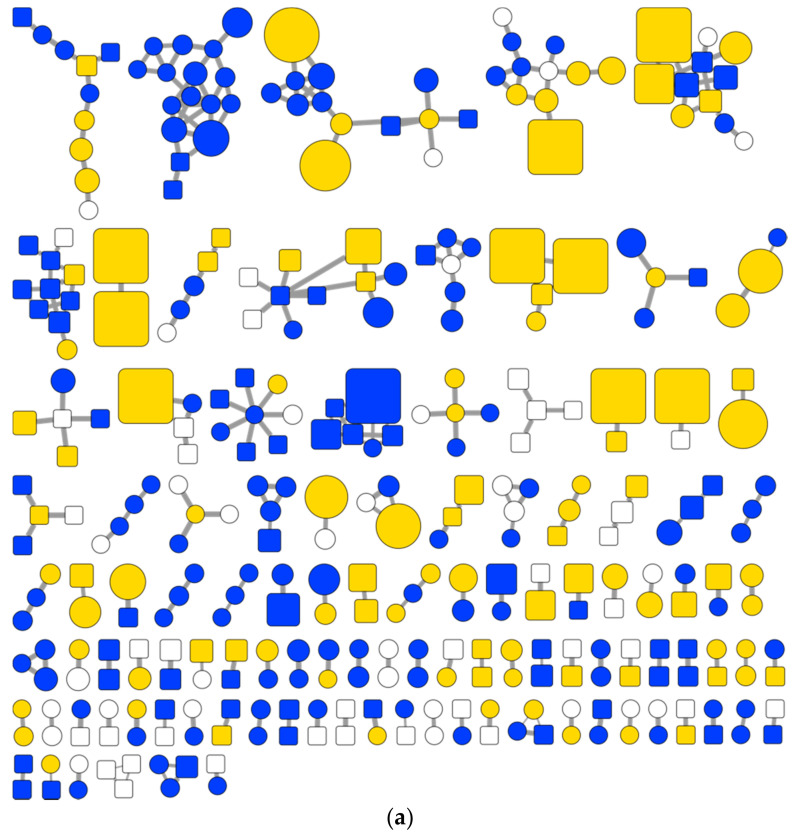

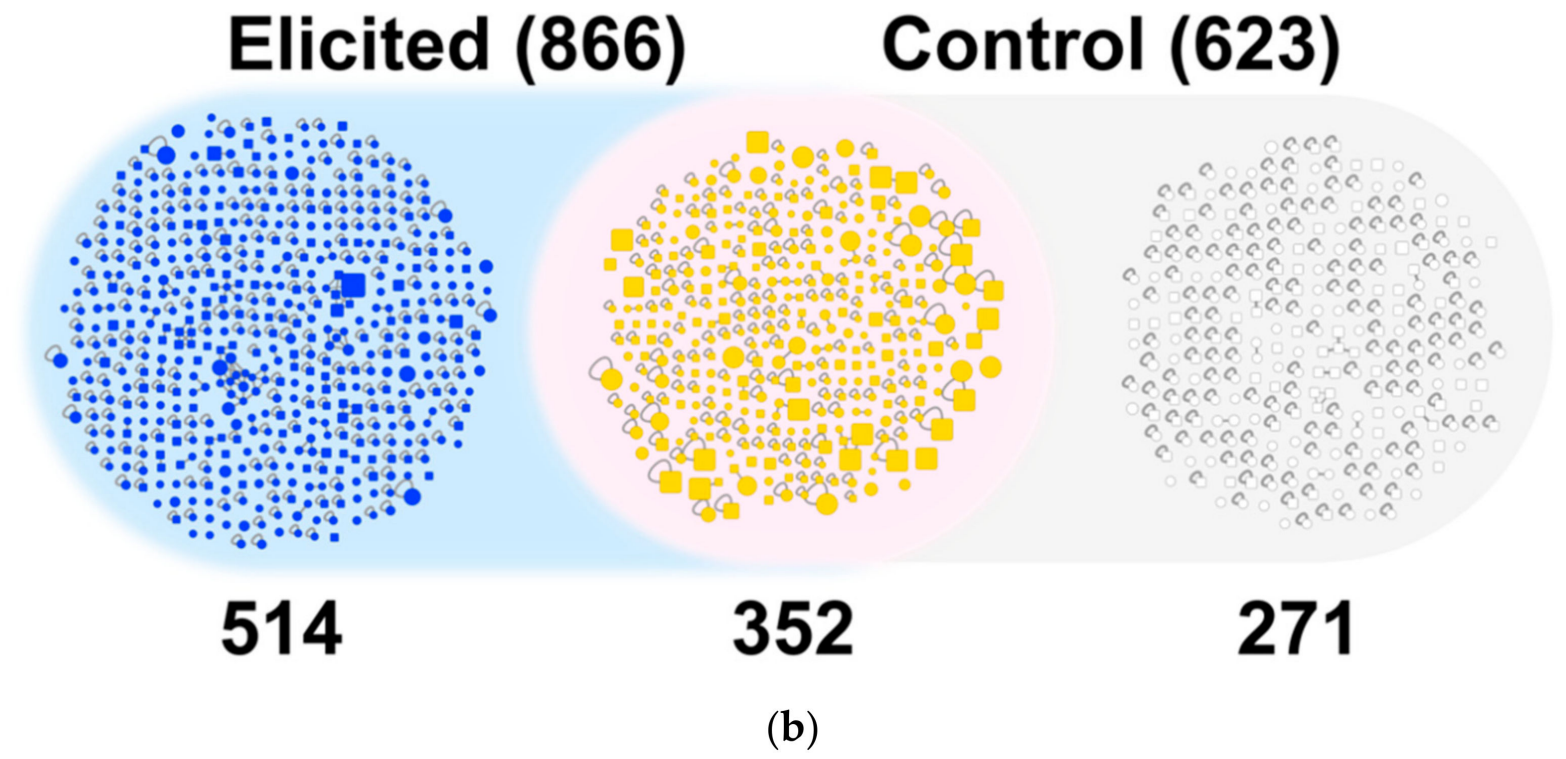
SSMN analysis of the [M + H]^+^ consensus spectra linked to bacterial metabolites detected in the EPfB chemical library shows the increment of chemical diversity promoted by bacterial cultivation in the presence of chemical elicitors. The network nodes (circles and squares) represent the [M + H]^+^ consensus spectra and the links connecting two nodes represent their spectra similarity score, ranging from 0 (totally dissimilar) to 1 (completely identical). The network is organized in spectra clusters by applying a spectra similarity threshold greater or equal to 0.6, limiting the number of neighbors of each node to 15 and limiting the clusters size to 200 nodes. (**a**) Network organization by the size of the spectra clusters, highlighting the larger clusters at the top of the network. The percent of ion contribution (peak area) of each growth condition to each node is denoted by the size of the node. Nodes are colored according to the sample of origin: exclusive to elicited growth conditions (blue), exclusive to control growth conditions (no elicitor added—in white), and common to both elicited and control conditions (yellow). The link’s width is proportional to the spectra similarity value between the two connected nodes. Self-loops (nodes with no connections) were excluded for better visualization. Nodes were further compared to MS/MS databases and annotated. The identified nodes are shown in squares. (**b**) Network organization by grouping the nodes detected preferentially under a given growth condition. Numbers indicate the total number of nodes in each group (including self-loops). The same color code in (**a**) is used.

The chemical diversity of the library was initially assessed by the visualization of the SSMN, which is based on the pairwise spectra similarity between the [M + H]^+^ consensus spectra extracted from the library (Figure 4). The molecular network is organized into clusters of similar spectra, each cluster representing chemically and structurally related compounds. Figure 4a is organized by cluster size, Figure 4b is organized according to the origin of the sample (elicited, control or both).

The analysis of the SSMN highlights the contribution of elicitation of bacterial cultures to the chemical diversity of the EPfB library. About 23% of the spectra clusters are composed only of nodes from elicited growth conditions (25/109 of the accounted clusters), potentially introducing new families of natural products (unique clusters) to the collection. Furthermore, elicited exclusive nodes are well distributed across the SSMN (Figure 4, in blue), covering 67% (73/109) of the spectra clusters, possibly contributing to chemical diversity around a given chemical scaffold. In combination with the common nodes (spectra found in both control and elicited conditions), the elicited samples cover about 94% (102/109) of the cluster found in the SSMN.

It is worth noting that there were clusters exclusively detected in the control conditions (without addition of any chemical elicitor), probably indicating that chemical elicitation also inhibited the production of some classes of bacterial metabolites, which were only detected in the control cultures. Seven small clusters (6% of total clusters) are composed only of nodes from non-elicited samples (Figure 4, in white).

Interestingly, a large cluster of possible biotransformation products of ampicillin was also found, composed of 16 nodes (large cluster colored blue in Figure 4a). Three bacterial strains were cultivated in the presence of ampicillin: LAMA915, LAMA585 and LAMA627; however, the biotransformation products were mainly found in LAMA915, which is a bacterial strain rich in beta-lactamases (GenBank assembly accession: GCA_001235865.1).

**Figure 5:**
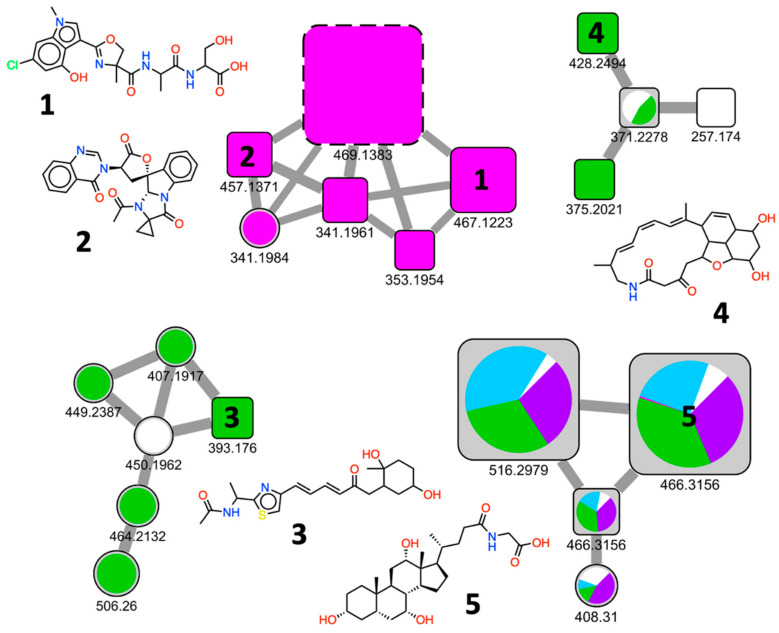
Chemical elicitation induced the production of rare and bioactive bacterial secondary metabolites. Selected clusters of consensus spectra were extracted from the SSMN of the EPfB library. JBIR-35 (1, *m*/*z* 467.1223) and tryptoquivaline K (2, *m*/*z* 457.1371) were coherently identified from a six-membered cluster elicited by kanamycin. In the same cluster, the major compound *m*/*z* 469.1383 (dashed lines) was denoted by a clear chlorine isotope pattern, incompatible with the proposed compound in the database, its identification being ignored from now on. Bacterial compounds leinamycin (LNM) K-3 (3, *m*/*z* 393.1760) and verticilactam (4, *m*/*z* 428.2494) are also highlighted in two clusters composed mainly by [M + H]^+^ consensus spectra derived from the EDTA elicited conditions. Glycocholic acid (5, *m*/*z* 466.3156), a compound found in marine bacteria, was found comprising a cluster of cholic acid derivatives in most of the growth conditions evaluated. Identified nodes are represented in squares; relative peak areas are represented in the pie-chart, colored according to the elicited growth condition: ampicillin (blue), kanamycin (magenta), procaine (purple), sodium butyrate (red), EDTA (green) and control conditions (white).

Four spectra clusters from the SSMN presented in Figure 4 were extracted to exemplify clusters of bioactive and rare natural products present in the library (Figure 5). Despite the identification given by the MS/MS databases used for comparisons, the data were also manually curated. This was done because some factors, such as coherence with the reported origin of the compounds (marine environment or bacterial natural products) and featured isotope occurrence (in case of halogens, for example) are not taken into account by the NP^3^ MS workflow at this point—or by any other MS/MS dereplication tool that we are aware of—when handling spectra identification. This may lead to erroneous spectra identification, or to difficulties in selecting the right compound from a list of possible identification candidates of a given MS/MS consensus spectrum.

From the selected clusters with identified compounds (Figure 5), JBIR-35 (1, *m*/*z* 467) [26], an indole-containing peptide, and tryptoquivaline K (2, *m*/*z* 457) [27], a quinazoline-containing indole alkaloid, were identified in a cluster from kanamycin-elicited samples of LAMA639 (Proteobacteria) culture. Both molecules were previously isolated from sponge-associated microorganisms. Interestingly, tryptoquivaline K (2) has a rare non-proteinogenic amino acid, 1-aminocyclopropane-1-carboxylic acid, uncommonly incorporated during the biosynthesis of the natural product [27]. A variety of biological results are reported in the literature for tryptoquivaline derivatives, including antifungal [28], antiviral [29] and NF-κB inhibitory activities [30].

The other two selected clusters showed the identification of LNM K-3 (3, *m*/*z* 393.1760), an intermediate in the biosynthesis of the antitumoral antibiotic leinamycin (LNM) from *Streptomyces* [31]; and verticilactam (4, *m*/*z* 428.2484), a macrolactam first isolated from *Streptomyces spiroverticillatus* JC-8444 [32]. Both compounds were detected in the EDTA elicited growth of B002_754.

A cluster of terpenes was selected, including glycocholic acid (5, *m/z* 466) identified from LAMA915 (Proteobacteria) samples. It was possible to note that the elicited conditions increased the detection of 5, especially by using EDTA and procaine elicitation. Bile acids, including glycocholic acid (a glycine-conjugated cholic acid), are essentially known for being products of cholesterol degradation in liver cells and its biotransformation plays a role in the regulation of the gut microbiome [33]. In the past decades, some reports have evidenced cholic acid derivatives biosynthesis by marine bacteria (*Myroides*, *Dokdonia*, *Polaribacter, Donghaeana*, *Maribacter*, *Hahella, Rhodococcus, Pseudovibrio*) [34,35,36]. Most recently, 5 was found in *Bacillus*, presenting antimicrobial activity against *P. aeruginosa* and *B. cereus* (MICs from 7 to 250 µg/mL) and *S. cerevisiae* (MICs = 15.6 µg/mL)—[37], highlighting its production by different bacterial groups, still showing antimicrobial activity towards other groups.

#### 2.2.4. Biological Relevance of the Library

**Figure 6:**
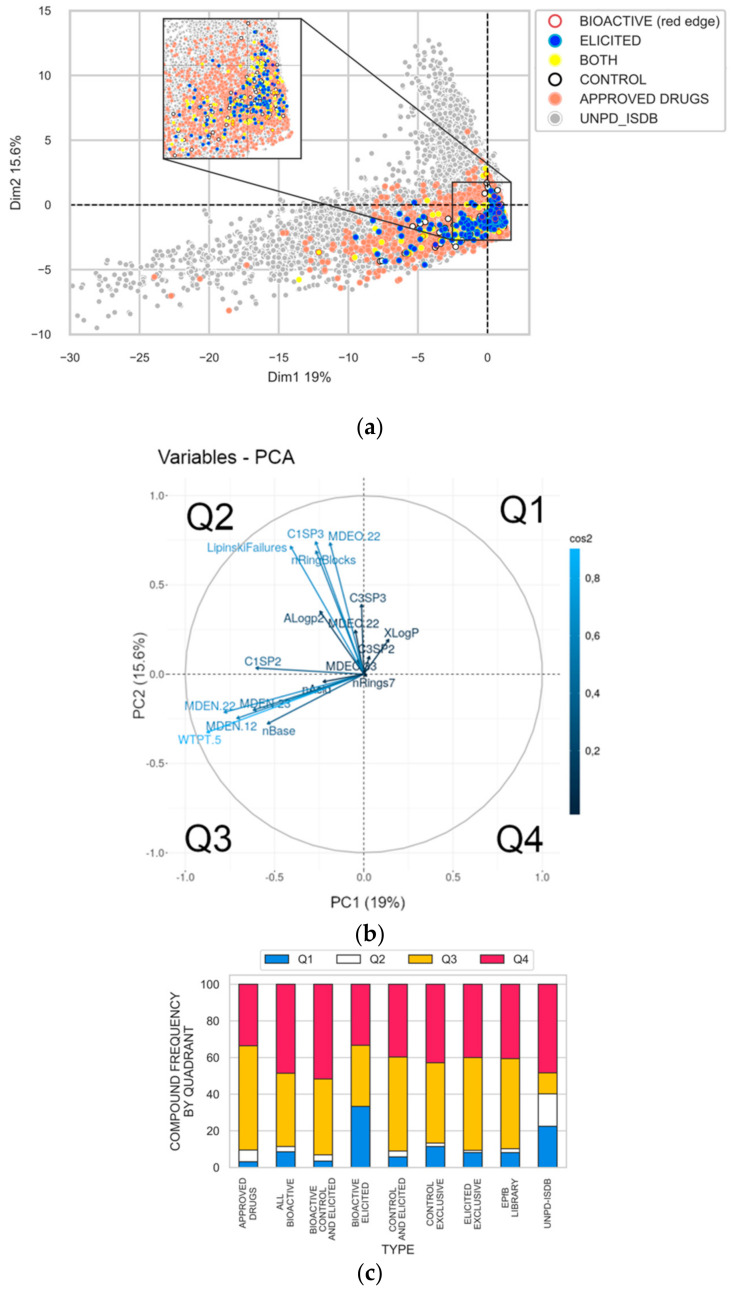
PCA based on molecular descriptors of compounds detected and identified in the EPfB library shows that the retrieved bacterial metabolites cover the chemical space of the approved drugs. (**a**) Chemical space coverage of identified compounds from consensus spectra exclusively detected in the following growth conditions: elicited (blue), controls (white) and both (yellow). Compounds found in the bioactive fractions are denoted by a red edge. For reference, compounds present in the UNPD-ISDB database (*n* = 208,009 natural products) and the DrugBank (subset of approved drugs, *n* = 2078 drugs) were also used in the PCA analysis and are shown in grey and salmon, respectively. A zoomed view at the central region of the PCA is shown in the inset for better visualization. (**b**) The PC1 and PC2 axes that separate the four quadrants (Q1 to Q4) are shown in black dotted lines. The main variables of contributing molecular descriptors are shown and labeled. (**c**) Relative frequency of compounds found in each quadrant by chemical collection/set.

The chemical analyses described above highlighted the diversity and potential novelty of the bacterial metabolites produced by deep-sea bacteria, which were further induced by chemical elicitors under laboratory growth conditions. The analysis of selected clusters and identified compounds (Figure 5) point to the relevant biological activity of these known natural products. We then questioned whether the global diversity of natural compounds obtained in this work might also hold biological significance, especially in the context of drug discovery. For this, we first used Principal Component Analysis (PCA) based on molecular descriptors, using the 424 consensus spectra pointed as [M + H]^+^ ions in our dataset that were identified by the GNPS (*n* = 12) and UNPD (*n* = 412) databases. We further used 208,009 natural products available in the UNPD-ISDB and 2078 approved drugs present in the DrugBank (https://www.drugbank.ca (accessed on 25 April 2019)) [38] as references for the chemical space of natural products and drugs, respectively. The molecular descriptors of each compound were calculated using CDK^®^ v2.0 package (https://cdk.github.io/ (accessed on 25 April 2019)) [39], including three groups: topological (*n* = 195), constitutional (*n* = 17) and geometric (*n* = 49). Only uncorrelated descriptors were considered to perform the analyses, resulting in the following 18 selected variables: ALogp2, C1SP2, C1SP3, C3SP2, C3SP3, LipinskiFailures, MDEC.22, MDEC.33, MDEN.12, MDEN.22, MDEN.23, MDEO.22, nAcid, nBase, nRingBlocks, nRings7, WTPT.5 and XLogP. The PCA plot resulted in a map corresponding to the chemical space covered by the identified compounds ([M + H]^+^ consensus spectra) from the EPfB library in comparison with the space covered by reported natural products (UNPD-ISDB) and approved drugs (DrugBank)—Figure 6.

The map is represented by four quadrants, sharing common topological and physicochemical features, and distributed according to the molecular descriptors (Q1: PC1, PC2 > 0; Q2: PC1 < 0, PC2 > 0; Q3: PC1, PC2 < 0; Q4: PC1 > 0; PC2 < 0)—Figure 6b. Natural compounds from UNPD-ISDB are spread across the chemical space, being concentrated mostly at Q4 (Figure 6c). The approved drugs (DrugBank) lie mostly in the lower quadrants (Q3 and Q4) of this PCA. There is an overlap of natural products and successful drug molecules, as already pointed by Dopson [40] and, more recently, by our group using the same type of analysis [1]. Importantly, ~20% of the natural products lie in quadrant Q2, where the descriptor “Lipinski Failures” is pointed, showing unwanted molecular features for orally administered drugs. Q2 is also represented by molecular descriptors, usually describing compounds rich in saturated carbons, such as C1SP3 and nRingBlocks (e.g., terpenes), and/or rich in oxygen and unsaturated carbons, such as MDEO.22 and C1SP2 (e.g., flavonoids and tannins). These classes of natural products are more often found in plants.

The EPfB library is almost devoid of compounds in Q2 and rather evidences compounds at Q3 and Q4 mainly, in a distribution across the chemical space that overlaps the distribution of the approved drugs (Figure 6a,c). Q3 is represented by molecular descriptors related to nitrogen atoms (MDEN.12, MDEN.22, MDEN.23 and WTPT.5) and charged compounds (nBase and nAcid). Small peptides are expected to be found at Q3. Additionally, the EPfB library presents about 40% of compounds at Q4, where bioavailable compounds are mostly expected, indicating that a considerable number of metabolites identified in our study display the molecular features of drug-like compounds. Chemical elicitation maintained the overall distribution of the identified compounds in the EPfB library (Figure 6c—“exclusive” bars).

The entire EPfB chemical library was further tested in four biological assays, being three cell-based assays—triple-negative breast cancer cells targeted for glutaminase inhibitors and Gram-positive/Gram-negative bacteria—and one enzymatic assay—proteasome inhibition (ChTL subunit). From the bioassays, 2 samples were selected and confirmed to display antibiotic activity (MIC < 16 µg/mL in Gram positive or Gram-negative bacteria; confirmed hit rate = 1%) and 2 samples were selected by inhibiting the enzymatic activity of the proteasome complex (IC_50_ < 40 µg/mL; confirmed hit rate = 1%). All the confirmed hits are represented by enriched fractions (Table 1).

The identified compounds present in the bioactive samples are highlighted by a red edge in Figure 6a. Most of the metabolites detected from the bioactive samples were found at Q4, followed by Q3 and Q1 (Figure 6c, “all bioactive” bars). Curiously, bioactive elicited fractions brought a high number of Q1 compounds (Figure 6c, “bioactive elicited” bars). Q1 is represented by the XlogP molecular descriptor, meaning more hydrophobic compounds. This is in accordance with the detection of antibiotic activity in the latest fractions (fractions 08 and 09) of the chromatographic series represented by LAMA639 and B002_754 samples (Table 1).

#### 2.2.5. Chemical Redundancy in the Relevant Biological Space

**Figure 7:**
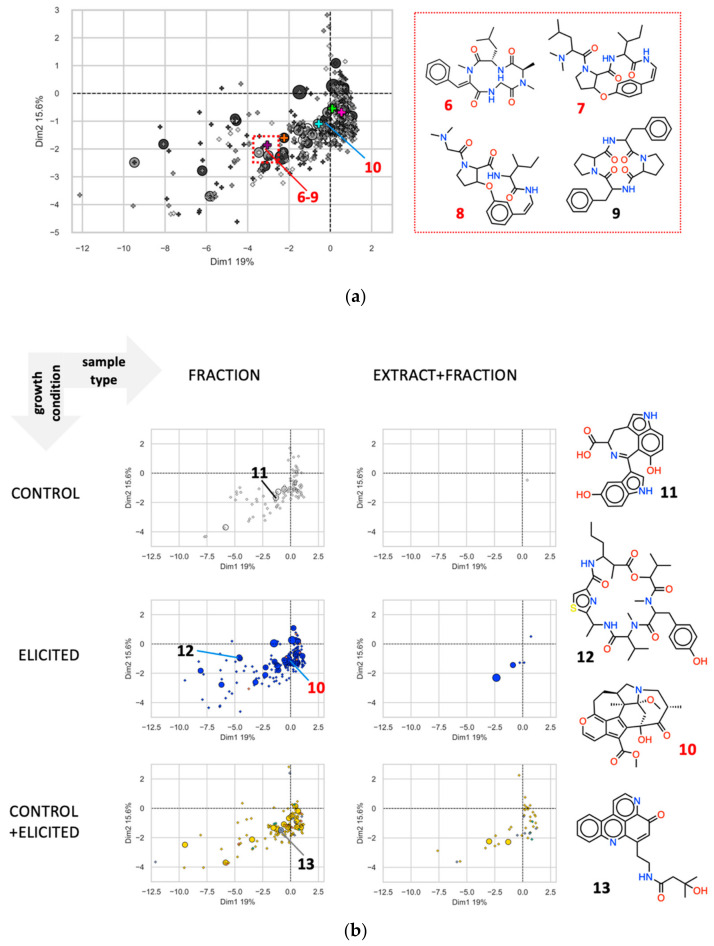
Expansion of the chemical space by inclusion of similar non-identified spectra and dissection of the chemical space by sample type and growth condition highlights the expression modulation of minor nitrogenated metabolites (Q3) and analogues by chemical elicitors. (**a**) A principal component analysis was performed based on molecular descriptors of identified [M + H]^+^ consensus spectra detected in this work. Each identified compound is represented by a cross in the plots and the circles denote the occurrence of non-identified analogous [M + H]^+^ consensus spectra in the collection. Circle size corresponds to the number of non-identified neighbors connected to the identified compound in the SSMN. Compounds 1–5 are highlighted purple, orange, cyan, green and magenta. (**b**) The PCA plot was split based on the cultivation method employed for bacterial cultures (growth condition: control (white), elicited (blue) and both (yellow)), and further by the sample type in which the spectra were found (sample type: chromatographic fraction (minor compounds) or both (major compounds)). Compounds derived from bioactive fractions are highlighted in grey (LAMA915), green (LAMA639) and red (B002_754). Representative compounds from the spectra databases used for metabolite identification, sharing spectra similarity to the EPfB [M + H]^+^ consensus spectra, were selected and are represented; 6: tentoxin; 7: mucronine J; 8: nummularine F; 9: cyclic tetrapeptide (PheProPhePro); 10: daphnicyclidin; 11: hyrtiazepine; 12: ulongamide D; and 13: cystodytin C. Numbers in red indicate natural products reported from organisms other than bacteria.

Next, we expanded the chemical space analysis of the EPfB library by introducing unidentified analogues of known natural products present in the spectra collection. For this, non-identified [M + H]^+^ consensus spectra that are linked in the SSMN to one of the 424 [M + H]^+^ consensus spectra identified in the MS/MS spectra databases used were also considered. This was done to further include part of the chemical novelty (the 713/1137 [M + H]^+^ consensus spectra not identified in databases), from which we could reference chemical descriptors by linking 57/713 of them to its most similar identified [M + H]^+^ consensus spectra in the chemical space (for more details see the Methods section). For a better visualization of this data, the PCA plot of the EPfB library is shown in Figure 7a, being further split by sample type and growth condition Figure 7b. The presence of potential analogues of the identified compounds are denoted by the size of the circle, landmarking the chemical space with the number of similar non-identified [M + H]^+^ consensus spectra directly linked to the identified node in the SSMN.

In the chemical space surrounding compound 1 (purple cross in Figure 7a at Q3) there are compounds identified from different SSMN clusters, which show spectra similarities to plant alkaloids mucronine J (7) [41], nummularine F (8) [42], tentoxin from endophytic fungi (6) [43] and cyclotetrapeptides from marine bacteria (9) [44]. Despite lying in the same chemical space, these compounds were identified from [M + H]^+^ consensus spectra from different SSMN clusters, showing that their spectra fragmentation is different, but their molecular features might be closely related. These cyclic peptides and alkaloids were found in all growth conditions. Cyclic peptides 6 and 9 were derived from B002_754 and B003_789 elicited by EDTA. These spectra were grouped into different clusters of the SSMN, with 6 in a cluster with 10 other nodes from the control and elicited conditions and 9 a sole spectrum in the SSMN. Compound 7 was identified from a [M + H]^+^ consensus spectrum only observed by B002_754 cultivation in the absence of chemical elicitors (control conditions), with one similar consensus spectrum added to this SSMN cluster in the EDTA condition. Compound 8, an analogue of 7, and similar [M + H]^+^ consensus spectra, were found in an independent cluster of the SSMN, being composed by nodes from LAMA585 (Firmicutes) and LAMA627 (Proteobacteria), in both control and elicited growth conditions.

The contribution of minor metabolites to the expansion of the chemical space (fraction exclusive—Figure 7b, left panels) should also be noted. Furthermore, similar compounds (non-identified, however similar to identified spectra in the SSMN) expanded the diversity around a given chemical scaffold, especially in the Q3 and Q4 regions of the chemical space (Figure 7b, represented by circles). Compound series at Q3 and Q4 seem to be upregulated by chemical elicitors, since most of the series were only detected in the elicited conditions (Figure 7b, in blue). A cluster of LAMA915 elicited by ampicillin and EDTA showed one [M + H]^+^ consensus spectrum with spectra similarities to compound 10, related to daphnicyclidin alkaloids isolated from the bark of *Daphniphyllum macropodum* [45]. This compound landmarks the chemical space of elicited minor compounds at the frontier of the Q3 and Q4 quadrants of the PCA. Ulongamide D (12) [46], a beta-aminoacid containing cyclodepsipeptides isolated from cyanobacteria, further expanded the chemical space of this set of samples to the left side of Q3, by showing spectra similarities with [M + H]^+^ consensus spectra found only in the elicited enriched fractions of B003_789.

In the control condition of B003_789, a small cluster represented by hyrtiazepine (11) is found (Figure 7b, in white), an indole alkaloid first isolated from a marine sponge with enzyme inhibition properties [19,47,48]. The expression of these compounds was inhibited by EDTA elicitation. Finally, cystodytin C (13) [49], an alkaloid first isolated from tunicates, showed spectra matches with one [M + H]^+^ consensus spectrum present in the bioactive fractions of LAMA915. This node is linked to a non-identified spectrum, being produced in both control and elicited growth conditions.

The production of known natural products as major compounds (found in both “extract and fraction column”—Figure 7b, right panels) was less affected by chemical elicitation. Only four identified major compounds were found in the elicited exclusive condition. These include the biotransformation products of ampicillin mentioned before (larger circle), the major compound of the elicited exclusive SSMN cluster from which compounds 1–2 were derived (Figure 5), and spectra matches to plant alkaloids (Q3) and hydrophobic diterpenes (Q1).

## 3. Discussion

Natural product libraries obtained from bacteria, fungi or plant are central for drug discovery [50]. Considering that secondary metabolites can be tightly regulated, laboratory culture conditions can be challenging to induce the expression of all or almost all metabolites encoded by the genome of bacteria or fungi, in order to obtain a representative library. There are numerous possibilities to be explored in culture conditions to mimic the natural environment, including pH, temperature, time of incubation, media composition and chemical elicitors [17]. The latter were explored in the present study, being systematically investigated by new methods of library preparation and analyses (Figure 1).

To obtain the EPfB chemical library presented in this work, a preliminary screening on agar plates was first established to select the most suitable chemical elicitor that would induce changes in colony morphology or populational variants, potentially mimicking the natural habitat and competitors of bacteria under laboratory conditions. Results obtained and presented in Figure 2 demonstrated how each strain growth was modulated by a specific elicitor. From this test, the most promising chemical elicitors were selected for large scale cultivation in liquid media aiming to induce the expression and to extract cryptic secondary metabolites. As shown, the chosen elicitors did in fact expand the chemical space of the library and gave access to a reservoir of small molecules that could not have been produced under normal culture conditions (please see also the Supplementary Materials for an analysis of chemical elicitors per bacterial strain). Bacterial cultivation on agar plates is a low-cost and accessible approach for screening multiple chemical elicitors in the process of preparing bacterial natural product libraries and should be used as a guide in selecting chemical elicitors for large-scale growth.

Bacterial crude extracts were obtained and fractionated by high performance liquid chromatography to construct the library, as enriched fractions are more suitable to perform high throughput biological screenings [51] and LC-MS/MS data collection [23,24]. Indeed, fractionation of the crude extracts evidenced about 90% of the bacterial metabolites (Figure 3c) and the biological activity (Table 1) detected from the samples comprising the EPfB chemical library.

The NP^3^ MS workflow was applied to extract the [M + H]^+^ MS/MS consensus spectra relative to bacterial metabolites, retaining the information of the sample of origin, its biological activity and chromatographic information. Finally, the GNPS and UNPD-ISDB databases were used to identify the [M + H]^+^ consensus spectra and the NP^3^ MS workflow selected the top results based on theMS/MS spectra matching scores. Improvements in MS/MS data mining tools are being pursued in the current literature [21,22,23,47]. Although none of them are currently perfect at retrieving the correct [M + H]^+^ ions or the correct identification of compounds from MS/MS spectra databases, untargeted metabolomics have increasingly proven to be a valuable asset for mining compound collections and comparing of biological groups.

The [M + H]^+^ consensus spectra retrieved from the EPfB library by the NP^3^ MS workflow were counted in each bacterial group, revealing that the elicitation of bacterial cultures had almost doubled (~45%) the number of compounds detected in the data set (Figure 3a). Analysis of the library’s [M + H]^+^ consensus spectra in comparison with databases revealed that most (63%) of these spectra were new to the MS/MS public collections accessed, half of them (49%) being only observed in the elicited growth conditions. Compound novelty was expected from the deep-sea bacteria collection used for the construction of the EPfB library. However, the use of the chemical elicitors further increased the number and the novelty of the metabolites produced ex situ, further highlighting the importance of chemical elicitation of bacterial cultures when prospecting novel bacterial natural products.

The SSMN (Figure 4) was constructed using the [M + H]^+^ MS/MS consensus spectra, giving a global visualization of the compound diversity introduced by chemical elicitation (Figure 4a). Clusters of compounds linked to bacterial metabolites were detected under specific growth conditions (Figure 4b). SSMNs have been extensively used in the last few years by the natural products community [23], and indeed represent a powerful tool to overview the chemical diversity contained, and to dereplicate unpurified natural product chemical samples. Groups of related compounds could be detected under specific growth conditions (Figure 5), highlighting rare bioactive compounds, such as tryptoquivaline K, the polyketide K-3, the macrolactam verticilactam and triterpenoid derivatives. Small clusters of non-identified spectra were also observed, potentially representing new series of compounds introduced by deep-sea bacteria and chemical elicitation, which might drive the discovery of novel natural products for drug development and chemical ecology.

Finally, the chemical space occupied by compounds identified from the EPfB library were compared to the natural products (UNPD-ISDB) and to the drug (Drug Bank, approved drugs) chemical spaces. The identified compounds cover the chemical space of drugs (Figure 6). Importantly, bacterial metabolites were shown to avoid the Lipinski’s failures region (Q2), which is partially occupied by known natural products. The bacterial collection further concentrated nitrogenated compounds (Q3), which are important molecular features of drugs. Overall, the compounds identified in the EPfB library span the chemical space of approved drugs, bringing chemical diversity in the relevant biological space. What is worth noticing is that the bioactive fractions derived from the biological screenings reported here concentrate compounds at Q3 and Q4, highlighting the importance of these regions of the chemical space also for in vitro bioactivity.

However, the abovementioned analyses were carried out with less than 40% of the bacterial metabolites identified from the EPfB library as MS/MS spectra database matches, from which chemical descriptors could be extracted. Part of the remaining [M + H]^+^ consensus spectra in our collection could be linked to at least one identified compound, covering 65% (71/109) of the SSMN clusters, further landmarking the chemical space covered by the EPfB library (Figure 7a). Non-identified analogues were concentrated at Q3 and Q4, most of the series minor compounds being modulated by chemical elicitors (Figure 7b).

The analyses presented in Figure 6 and Figure 7 complemented well the SSMN evaluation, grouping the identified compounds and their potential analogues by chemical descriptors, independent of their spectra similarities. This assisted the characterization of the chemical space covered by the EPfB library. In particular, Q3—a diverse and relevant chemical space of drugs—shows nitrogen-rich compounds of moderate molecular complexity, derived from spectra matches from fractions of different bacteria, especially in the elicited and control exclusive growth conditions. Since this region concentrates small modified peptides, it is possible that NRPS and mixed NRPS biosynthetic pathways are directly affected by chemical elicitors.

The additional analyses of the EPfB library per strain, detailed in the Supplementary Materials, further revealed that all the chemical elicitors used modulated the production of metabolites in all bacterial strains. The effect of a given chemical elicitor appeared to be strain dependent, it being the contribution of metabolites induced by the chemical elicitor variable according to the bacterial strain. The bacterial strains further responded differently to the distinct chemical elicitors used, with a particular chemical elicitor being more effective in introducing non-redundant and novel metabolites by a given strain. Therefore, testing and selecting more than one chemical elicitor for a given bacterial strain growth under laboratory conditions is important towards the production of the maximum number of metabolites possible for a particular strain.

## 4. Materials and Methods

LAMA collection: Bacterial strains were isolated from marine sediment or water collected at up to 11,000 m depth at Walvis Ridge Sector [52], South Equatorial MAR Sector [53] or Rio Grande Rise. Bacterial isolates were grown in TSA (Tryptic Soy Agar) or MA (Marine Agar), 28 °C, 24 h for reactivation.

Elicitation tests (small scale—solid media): bacteria were inoculated in 5 mL TSB (Tryptic Soy Broth), MB (Marine Broth) or YEME (Yeast Maltose Extract) media, at 28 °C, 200 rpm, for 2 h, 17 h and 24 h. For each time point, 100 µL were aliquoted for measurement of OD_600_ measurement; 1 mL from each preculture was centrifuged at 13,000 rpm for 1 min and the pellet was resuspend in 100 µL of supernatant, plated onto selected solid media and incubated at 28 °C, for 16 h. Then, 5 µL drops of the chemical elicitors were directly applied on each culture using an automated pipette (Eppendorf Xplorer 8 channels). For this, stock solutions of each chemical elicitors, prepared at different concentrations, were used. The cultures were incubated at 28 °C for 24 h. Following the incubation time, each culture plate was visually inspected in the region corresponding to each elicitor drop and classified accordingly to the changes in colony morphology in this spot (vide Figure 2).

Elicitation—large scale (liquid media): Selected strains were inoculated in 5 mL TSB (Tryptic Soy Broth—LAMA585, LAMA627, LAMA639, B003_789, LAMA627, B004_912), MB (Marine Broth—LAMA915) or YEME (Yeast Maltose Extract—B002_754) at 28 °C, 200 rpm, for 2 h, 17 h or 24 h. Each preculture (5 mL) was inoculated at 400 mL of selected media and incubated at 28 °C, 200 rpm. Then, elicitors were added at a previously established concentration on solid media experiments. Next, 40 g of Amberlite XADHP: Diaion HP-20 (1:1) resins were added to the liquid cultures after 24 h of bacterial growth in the presence of the elicitor, this being maintained for another 24 h for metabolite adsorption to the resin. The culture was filtered (membrane 0.22 µm) and washed with 400 mL Milli-Q water and 200 mL of methanol was added. After 48 h, the methanolic extract was separated by filtration. For second elution, 200 mL of methanol was added to the resin and after 1 h the suspension was sonicated for 15 min followed by filtration. Both elutions were combined, and the solvent was evaporated to completion in vacuo.

Pre-fractionated library preparation: Previously obtained extracts were resuspended in H_2_O:CH_3_OH:DMSO (2:1:1) to a 60 mg/mL concentration. Samples were fractionated by injection of 5 mL aliquots (~300 mg crude extract) onto a SunFire C18 OBD reversed-phase prep column (10 μM, 30 mm × 100 mm) attached to a compatible pre-column and coupled to an AutoPurification HPLC-PDA (Waters, Milford, MA, USA) system. A solvent system of water (A) and methanol (B) and the following analytical method was used: 12 mL/min flow rate; 0−21 min 10% B to 50% B; 21−27 min, 50% B to 100% B; 27−30 min, 100% B; 30−40 min for column equilibration. Fractions were collected in 36 mL/50 mL tubes. A solvent from each of 9 enriched fractions was evaporated to completion in vacuo. Samples were resuspended in DMSO to a final concentration of 10 mg/mL by vortexing and 5 min sonication (Easy-Elmasonic). The chemical samples were aliquoted (25 µl at 10 mg/mL) in 384-well plates, using half a row for each chromatographic series, containing the crude extract and the 9 fractions, totaling 18 crude extracts and 162 fractions. This layout was chosen to systematically evaluate the enrichment of metabolites by pre-fractionating the crude extracts, as previously suggested by Wagenaar [51] and Grkovic et al. [54] Daughter plates were prepared at 1 mg/mL and 0.1 mg/mL concentrations for the bioassays.

LC-MS/MS data collection: For chemical evaluation, a 2 µL aliquot of extract was injected into a BEH C18 reversed-phase column (1.7 μM, 2.1 mm × 100 mm) attached to a compatible pre-column and analyzed with an Acquity H-Class UPLC (Waters, Milford, MA, USA) system coupled to a Bruker Impact II UHR-ESI-QqTOF mass spectrometer (Bruker Daltonics, Billerica, MA, USA). A solvent system of water (A), acetonitrile (B) and 2% formic acid (C) was made and the following analytical method was used: 0.5 mL/min flow rate; column temperature 40 °C; 0−1 min, 5% B; 1–7 min, 5% B to 35% B; 7−11 min, 35% B to 95% B; 11−13 min, 95% B; 13−15 min for column equilibration on initial phase. C was kept at 5% constantly (final concentration of 0.1%). The mass spectrometer worked in positive ion mode scanning mass from 30 to 1800 Da range, acquisition rate of 8 Hz. End plate offset = 500 Volts (V); V_cap_ 4500 V; nebulizer 4.0 bar; drying gas (N_2_) flow 10 L/min; drying gas temp 200 °C), followed by an MS/MS scan for the most intense ions in a cycle time of 1 s, absolute threshold (per 1000 sum.) of 1500 cts. As MS^2^ rules, mass ratios (*m/z*) below 200 Da were excluded, and the “active exclusion” function was enabled (precursor ions with more than 3 spectra had their fragmentation blocked for 0.3 min or until the “current intensity/previous intensity” ratio was greater or equal to 1.8). Each run was automatically calibrated using HCOONa (10 mM) and calibrated spectra were converted in mzXML files through Data Analysis 4.0 and included bio tools CompassXport.

Data processing and analysis using the NP^3^ Mass Spectrometry (MS) Workflow: This workflow is an in-house collection of scripts to enhance untargeted metabolomic research focused on drug discovery with optimizations towards natural products. The beta version is freely available in our repository (https://bitbucket.org/cnpemlqpn/np3_ms_workflow/src/master/ (accessed on 18 December 2020)), with a detailed manual and a command line interface. The NP^3^ MS workflow uses a metadata file manually constructed by the user to describe the LC-MS/MS files used in the job, the files types, their grouping information and bioactivity scores. For this job a metadata file with 229 mzXML files was compiled and the samples codes summarized the EPfB chemical library samples. The sample codes tracked information on the bacterial strain of origin, the growth conditions and the sample type. The bacterial strain code was the prefix of the samples code, which was followed by the growth condition with the name of the chemical elicitor, and the sample type as the suffix: 00 for crude extracts and 01 to 09 for fractions. As an example, L585_BUT50_09 is the nineth fraction of LAMA585 crude extract derived from LAMA585 growth in the presence of butyrate 50 µM; L585_00 refers to the crude extract of LAMA585 grown in the control condition (no chemical elicitor added); L585_07 is the seventh chromatographic fraction of LAMA585 crude extract in the control growth condition. Chromatographic blanks were annotated as BLANK, chemical elicitors as STANDARD and culture media as BED.

The NP^3^ MS workflow (command “run”) processed the input EPfB’s mzXML files from the metadata file, and returned the following main files as outputs: (i) the Mascot Generic Format (MGF) file, containing the collection of consensus spectra extracted; (ii) the clean count table “*_peak_area_clean_annotated.csv”, with the consensus spectra’s quantification in each sample of origin (tracked by the sample codes) and identifications against the UNPD in-silico database; (iii) the edges file “*_molecular_networking_sim_06_topK_15_maxComponent_200.selfloop”, with the connections of the raw Spectra Similarity Molecular Network (SSMN); (iv) the edges file “*_molecular_networking_annotations.selfloop”, with the connections of the Ionization Variant Annotation Molecular Networking (IVAMN); and v) the table “*_molecular_networking_annotations_attributes_protonated_representative.csv”, resulting from a link analysis of the IVAMN with the suggestions of putative [M + H]^+^ consensus spectra candidates (column “protonated_representative” equals 1), to represent the real metabolites of the library.

The nodes of the IVAMN are the collection of consensus spectra present in the “*_peak_area_clean_annotated.csv” and the links of this network connects two consensus spectra that have a chemical annotation. Adducts, neutral losses, multiple charge, dimers/trimers, isotopes and in-source fragmentation are the ionization variants considered, based on chemical rules and numerical equivalences. The links have a direction, pointing from the consensus spectra considered as an ion variant to the consensus spectra considered as a putative [M + H]^+^ candidate in the respective annotation (e.g., [M+Na]+ -> [M + H]^+^).

Experimental MS/MS database dereplication with GNPS library: For including GNPS library identifications, the resulting MGF consensus spectra list from the NP^3^ MS workflow was used as input in the GNPS website aiming dereplication of the EPfB library against the experimental MS/MS collection of GNPS [23]. The GNPS spectra were filtered in the same manner as the input data. All matches kept between network spectra and library spectra were required to have a score above 0.65 and at least 6 matched peaks. The identifications from GNPS were combined in the “*_peak_area_clean_annotated.csv” table (NP^3^ output) using the NP^3^ MS workflow command “gnps_result “.

Selection of the [M + H]^+^ consensus spectra relative to bacterial metabolites: The IVAMN was used to remove the ions from the chromatographic blanks, chemical elicitors, and culture media samples. For this, the [M + H]^+^ table (output “v” from NP^3^), the “*_peak_area_clean_annotated.csv” with GNPS results added, and the IVAMN edges file (output “iv” from NP^3^) were visualized in Cytoscape v3.8.0. Then, spectra clusters relative to blank or culture media were removed by manually filtering all the clusters that had at least one node present in BLANK, STANDARD or BED samples. With this, only the [M + H]^+^ consensus spectra remaining in the clean IVAMN, which are the most possible real bacteria metabolites, were selected and exported in a CSV table named “IVAMN_clean_protonated.csv”, together with the quantifications and identifications that were present in clean count table.

SSMN construction and analysis: The raw SSMN returned by the NP^3^ MS workflow (output “iii” from NP^3^) was visualized with Cytoscape v3.8.0. Next, the “IVAMN_clean_protonated.csv” (bacterial metabolites list and attributes) was imported and used to filter only the nodes present in this list. As a result, the filtered SSMN nodes contained only the [M + H]^+^ consensus spectra relative to bacterial metabolites in the EPfB library. The SSMN edges file was exported as “SSMN_filtered_protonated.selfloops” for further use in the workflow. This filtered SSMN and its attributes from the “IVAMN_clean_protonated.csv” was used in the analyses presented in this work.

Cytoscape regex filter, based on the sample code format described above, were used to select and distinguish (colors, formats, etc) the network nodes according to the sample of origin, quantity in the sample of origin, chemical elicitation and sample type. The GNPS and UNPD identifications columns were used to select and distinguish the identified [M + H]^+^ consensus spectra relative to bacterial metabolites.

Spectra count: The quantification and identification columns of the “IVAMN_clean_protonated.csv” were used to count the bacterial metabolites in the different samples of the EPfB library and to prune novel compounds. Python scripts, helped by pandas library and matplotlib, were used for retrieving the data and plotting the bar charts.

Euler diagrams: A Python script counted all intersections, unions, and disjoints quantifications by sample and by database identification also using the “IVAMN_clean_protonated.csv” as input. The resulting numbers were the input for eulerr R library version 6.1.0 (https://github.com/jolars/eulerr (accessed on 25 April 2019)). Eulerr generates area-proportional Euler diagrams displaying set relationships.

Chemical space mapping: The evaluation of the chemical space was performed by Principal Component Analysis (PCA) using molecular descriptors extracted from each identified compound. The “IVAMN_clean_protonated.csv” file, which also contains the database identifications, was used to retrieve the smiles of the compounds suggested by databases as spectra matches to the EPfB library metabolites. Only the top-ranked smile string, based on MQscore, were used in the PCA analyses.

Chemical spaces were constructed based on the approach used in [1] with minor modifications. The compounds deposited at the UNPD-ISDB and at the Drug Brank (set approved drugs) were used, along with the compounds identified from the elicited pre-fractionated bacterial (EPfB) chemical library reported here. The calculation of all molecular descriptors for each dataset was preformed using the CDK^®^ v2.0 package (https://cdk.github.io/ (accessed on 25 April 2019)) [39]. Duplicates were removed. The following descriptors were used: topological (*n* = 195), constitutional (*n* = 17) and geometric (*n* = 49) and only uncorrelated descriptors were considered for the PCA analysis, chosen through a Spearman’s correlation matrix. The descriptors used in the PCA were: ALogp2, C1SP2, C1SP3, C3SP2, C3SP3, LipinskiFailures, MDEC.22, MDEC.33, MDEN.12, MDEN.22, MDEN.23, MDEO.22, nAcid, nBase, nRingBlocks, nRings7, WTPT.5 and XLogP.

Non-identified metabolites, that showed spectra similarity with identified ones, were introduced to the chemical space map using the information of spectra similarity present in the links of the filtered SSMN (file: “SSMN_filtered_protonated.selfloops” mentionated above).

The same PCA constructed for known and identified compounds was used; however, the representation of the points containing non-identified similar compounds was modified as follows: identified compounds were represented in the chemical space by a cross, whereas compounds bringing non-identified similar spectra were further represented by circles, in which the size of the circle was weighted according to the number of non-identified similar spectra linked to a given identified compound of the EPfB chemical library. In this way, a non-identified spectrum was added to the chemical space, being represented by its most similar identified neighbor. The smaller circle represents one similar compound connected and the bigger one represents two. Redundancies to other links were avoided by only considering directly connected nodes, a similarity cut-off of cosine ≥0.6 and further filters as explained in Figure 4.

Biological assays: The 180 samples of the EPfB library (18 crude extracts and 162 fractions) were screened in four bioassays. Proteasome screening was performed as previously reported [55]. Human 20S proteasome was obtained from Enzo Life Sciences and LLVY-AMC fluorogenic substrate (specific for the ChTL subunit) from Boston Biosciences. Gram-positive and Gram-negative antibiotic screening was performed following the protocols used in reference [56,57]. [57] Triple-negative breast cancer cell antiproliferative activity was assessed using sub-cell lines generated either by the transduction with lentivirus expressing shRNA against glutaminase mRNA or mock lentivirus (expressing shRNA against green fluorescent protein (GFP) gene) (manuscript in preparation).

## 5. Conclusions

In conclusion, the EPfB chemical library represents a chemically diverse collection for drug discovery efforts. This library was produced by uncommon bacteria, from which metabolites were induced by chemical elicitation and evidenced by fractionation of the crude extracts. This pilot work pinpoints strategies for constructing and evaluating larger and chemically diverse bacterial natural product libraries, including extreme environment bacteria, such as the LAMA deep-sea bacteria collection. The reported methods and findings will assist further work towards the identification of novel bacterial secondary metabolites in natural product-based drug discovery pipelines.

## Figures and Tables

**Table 1:** Hits selected from the biological screenings carried out with the prepared elicited deep-sea bacterial chemical library. Crude extract samples from which the chromatographic fraction was obtained were included for reference.

Bioassay	Source Bacteria	Growth Condition	Bioactive Sample	Bioactivity	
				*E. coli*	*B. subtilis*
bacterial growth inhibition	LAMA639	kanamycin	Crude extract	64 ^1^ (74%) ^2^	n.d. ^1^(<40%) ^2^
Bacterial growth inhibition	LAMA639	kanamycin	Fraction 08	4 ^1^ (100%) ^2^	2 ^1^ (99%) ^2^
bacterial growth inhibition	B002_754	EDTA	Crude extract	n.d.^1^(<40%) ^2^	n.d. ^1^ (48%)
bacterial growth inhibition	B002_754	EDTA	Fraction 09	n.d. (<40%) ^2^	8 (99%) ^2^
				*ChTL h20S*	
proteasome	LAMA915	control	Crude extract	n.d. ^3^ (0%) ^4^	
proteasome	LAMA915	control	Fraction 06	25 ^3^ (55%) ^4^	
proteasome	LAMA915	procaine	Crude extract	n.d. ^3^ (0%) ^4^	
proteasome	LAMA915	procaine	Fraction 06	16 ^3^ (0%) ^4^	

^1^ MIC (µg/mL), ^2^ percent growth inhibition at 20 µg/mL of Gram- (*E. coli*) and Gram+ (*B. subtilis*) bacteria. ^3^ IC_50_ (µg/mL), ^4^ percent enzyme activity inhibition at 40 µg/mL of the ChTL subunit of the human 20S proteasome. n.d.: not detected.

## Data Availability

GNPS Molecular Networking data is available at: https://gnps.ucsd.edu/ProteoSAFe/status.jsp?task=8cfc13c1864a4a20b14f342930a770b5 (accessed on 21 July 2020).

## Supplementary Materials

The following are available online at https://www.mdpi.com/2218-1989/11/2/107/s1. The analyses of the EPfB library per strain are detailed in the Supporting Information, with 8 Tables and 10 Figures.
