# Supplementary material for: Chemical Elicitors Induce Rare Bioactive Secondary Metabolites in Deep-Sea Bacteria under Laboratory Conditions
Source: Metabolites. 2021 Feb 12;11(2):107. doi: 10.3390/metabo11020107 (PMC7918856; doi:10.3390/metabo11020107)
Supplement: Supplementary file 1 [file metabolites-11-00107-s001.pdf]

Supplementary Materials

Chemical elicitors induce rare bioactive secondary metabolites in deep-sea bacteria under laboratory conditions

Rafael de Felício [1], Patricia Ballone [1,2], Cristina Freitas Bazzano [1,3], Luiz F. G. Alves [1], Renata Sigrist [1], Gina Polo Infante [1], Henrique Niero [1,2], Fernanda Rodrigues-Costa [1,2], Arthur Zanetti Nunes Fernandes [1,2], Luciane A. C. Tonon [1], Luciana S. Paradela [1], Renna Karoline Eloi Costa [1], Sandra Martha Gomes Dias [1], Andréa Dessen [1,4], Guilherme P. Telles [3], Marcus Adonai Castro da Silva [5], Andre Oliveira de Souza Lima [5] and Daniela Barretto Barbosa Trivella [1,*]

[1] Brazilian Biosciences National Laboratory (LNBio), National Center for Research in Energy and Materials (CNPEM), 13083-970, Campinas, SP, Brazil.

[2] Institute of Biology, University of Campinas (UNICAMP), 13083-862, Campinas, SP, Brazil.

[3] Institute of Computing (IC), University of Campinas (UNICAMP), 13083-852, Campinas, SP, Brazil.

[4] Univ. Grenoble Alpes, CNRS, CEA, Institut de Biologie Structurale (IBS), F- 38000, Grenoble, France.

[5] School of Sea, Science and Technology, University of Vale do Itajaí (Univali), 88302-202, Itajaí, SC, Brazil.

* Correspondence: daniela.trivella@lnbio.cnpem.br; Tel.: +55-19-3517-5055

This set of Supplementary Materials brings analyses of the metabolites detected from the EPfB pilot library, with focus on comparing the 7 different strains used in the preparation of the library. It was analyzed: i) the metabolite contribution per strain and chemical elicitor; ii) the effect of chemical elicitors on the metabolome of each strain; iii) novel compounds introduced in the EPfB library by each strain, regarding metabolite identification against MS/MS spectra databases; iv) the chemical diversity introduced by each bacterial strain as for SSMN analyses; and vi) the contribution of each bacterial strain to the EPfB library chemical space coverage.

Table of Contents

Metabolite distribution per strain and chemical elicitor 4

Effect of chemical elicitors on the metabolome of each strain.................................. 5

Novel compounds introduced by each strain.. 8

Metabolic exclusivity and redundancy among the bacterial strains 10

Novelty of the strain exclusive metabolites 13

Chemical diversity introduced by each bacterial strain 15

Chemical space coverage of the strain exclusive upregulated metabolites 17

General conclusion of the strain specific analyses 18

List of Tables

Table S1. Distribution of metabolites detected from samples derived from each of the 7 bacterial strains used for the preparation of the EPfB library. 4

Table S2. Percent of metabolites detected in each strain relative the total EPfB library and to each growth condition...4

Table S3. Distribution of metabolites detected exclusively in the elicited conditions (upregulated) of the 7 bacterial strains used for the preparation of the EPfB library. 6

Table S4. Distribution of novel metabolites produced by each bacterial strain and the influence of chemical elicitation..8

Table S5. Distribution of exclusive metabolites *per* strain in comparison to the whole EPfB library. 10

Table S6. Distribution of redundant metabolites across the 7 bacterial strains used for the preparation of the EPfB library. 11

Table S7. Distribution of exclusive and novel metabolites across the 7 bacterial strains used for the preparation of the EPfB library. 12

Table S8. Common metabolites for different strains...14

List of Figures

Figure S1. Distribution of metabolites detected in each strain...5

Figure S2. Euler diagrams of the 7 bacterial strains that compose the EPfB chemical library, grouping the metabolites detected in each strain by the chemical elicitor used in the bacterial cultures. 6

Figure S3. Distribution of elicited metabolites in different strains. 7

Figure S4. Distribution of novel metabolites detected in each strain.................................9

Figure S5. Euler diagrams of the metabolites produced by the different strains used for the construction of the EPfB library. 10

Figure S6. Metabolite redundancy among the 7 strains that compose the pilot EPfB library...12

Figure S7. Metabolites that were exclusively detected in one of the 7 strains that compose the pilot EPfB library...13

Figure S8. SSMN of the EPfB library colored by the producing bacteria. 16

Figure S9. SSMN of the metabolites that were strain exclusive and upregulated by chemical elicitors...17

Figure S10. Chemical space of the upregulated and strain exclusive bacterial metabolites in the EPfB library. 18

Metabolite distribution per strain and chemical elicitor

First, we analyzed the distribution of the detected metabolites in each individual bacterial strain (**Table S1**). On average, 229 metabolites were detected in the samples derived from each strain, with maximum of 316 (LAMA585) and minimum of 71 (B004_912) metabolites detected per strain. Each bacterial strain contributed on average to 20.1% to of the metabolites contained in the EPfB library [maximum of 27.8% (LAMA585), minimum of 6.2% (B004_912)] - **Table S1** and **Figure S1** (black bars).

Table S1. Distribution of metabolites detected from samples derived from each of the 7 bacterial strains used for the preparation of the EPfB library.

The number of potentially novel metabolites (not identified against MS/MS spectra databases) found in each strain is denoted in parenthesis.

Strain	Total (novel)	Both (novel)	Control exclusive (novel)	Elicited exclusive (novel)
**LAMA585**	316 (217)	97 (72)	41 (26)	178 (119)
**LAMA627**	204 (144)	23 (16)	56 (41)	125 (87)
**LAMA639**	263 (120)	89 (38)	107 (48)	67 (34)
**B002_754**	273 (151)	25 (12)	111 (67)	137 (72)
**B003_789**	214 (155)	23 (16)	66 (41)	125 (98)
**B004_912**	71 (50)	32 (22)	21 (11)	18 (17)
**LAMA915**	260 (146)	94 (39)	27 (14)	139 (93)
average	***229 (140)***	***55 (31)***	***61 (35)***	***113 (74)***

Table S2. Percent of metabolites detected in each strain relative the total EPfB library and to each growth condition.

Percentages were calculated using the numbers shown in Table S1 (please see footnote for detaisl). Each cell in the table is colored according to its rank relative to the maximum value observed in each column (green scale: highest value in the column in green, lowest value in the column in white).

Strain	% overall contribution to the EPfB library[a]	% not influenced by chemical elicitors[b]	% downregulation[c]	% upregulation[d]
**LAMA585 (AMP, EDTA, BUT)**	27.8	30.7	13.0	56.3
**LAMA627 (AMP)**	17.9	11.3	27.5	61.3
**LAMA639 (KAN)**	23.1	33.8	40.7	25.5
**B002_754 (EDTA)**	24.0	9.2	40.7	50.2
**B003_789 (EDTA)**	18.8	10.7	30.8	58.4
**B004_912 (EDTA)**	6.2	45.1	29.6	25.4
**LAMA915 (AMP, EDTA, PRO)**	22.9	36.2	10.4	53.5
average	***20.1%***	***25.3%***	***27.5%***	***47.2%***

[a] total number of metabolites detected in a given strain divided by the total number of metabolites in the EPfB library (n=1,137).

[b] detected in both control and elicited conditions. Percentage calculated using the total number of metabolites detected in both control and elicited growth conditions of a given strain divided by the total number of metabolites this bacterial strain produced.

[c] detected in control conditions only (control exclusive). Percentage calculated using the total number of metabolites detected exclusively in the control growth condition of a given strain divided by the total number of metabolites this bacterial strain produced.

[d] detected in elicited conditions only (elicited exclusive). Percentage calculated using the total number of metabolites detected exclusively in the elicited growth condition of a given strain divided by the total number of metabolites this bacterial strain produced.

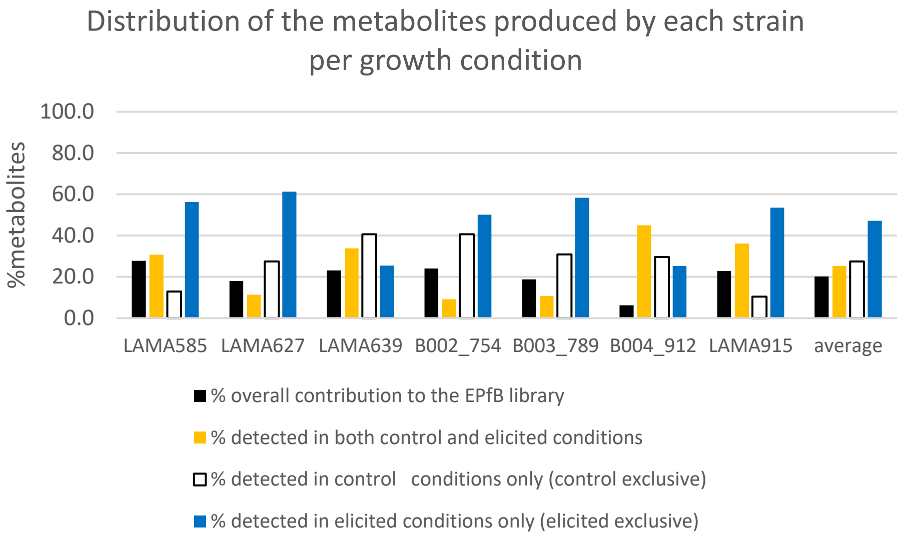

Figure S1. Distribution of metabolites detected in each strain.

Distribution of the metabolites per strain in each growth condition: common to both control and elicited (yellow), detected exclusively in the elicited growth conditions (blue) or exclusively in the control growth condition (white). Data are plotted according to Table S2. The percent of metabolite contribution of the strain to the EPfB library is also plotted for reference (black).

Effect of chemical elicitors on the metabolome of each strain

The chemical elicitors used in this work influenced metabolite production in the bacteria strains (please see **Figure S2** for an overview). Most of the elicited conditions used upregulated the production of bacterial metabolites (**Table S2** and **Figure S1**, blue bars). On average, about half (47.2%) of the metabolites produced by each strain were upregulated, being exclusively found in the elicited conditions [maximum 61.3% in LAMA 627 (ampicillin), minimum 25.4% in B004_912 (EDTA)]. Therefore, the chemical elicitors used increased the universe of metabolites a given strain can produce.

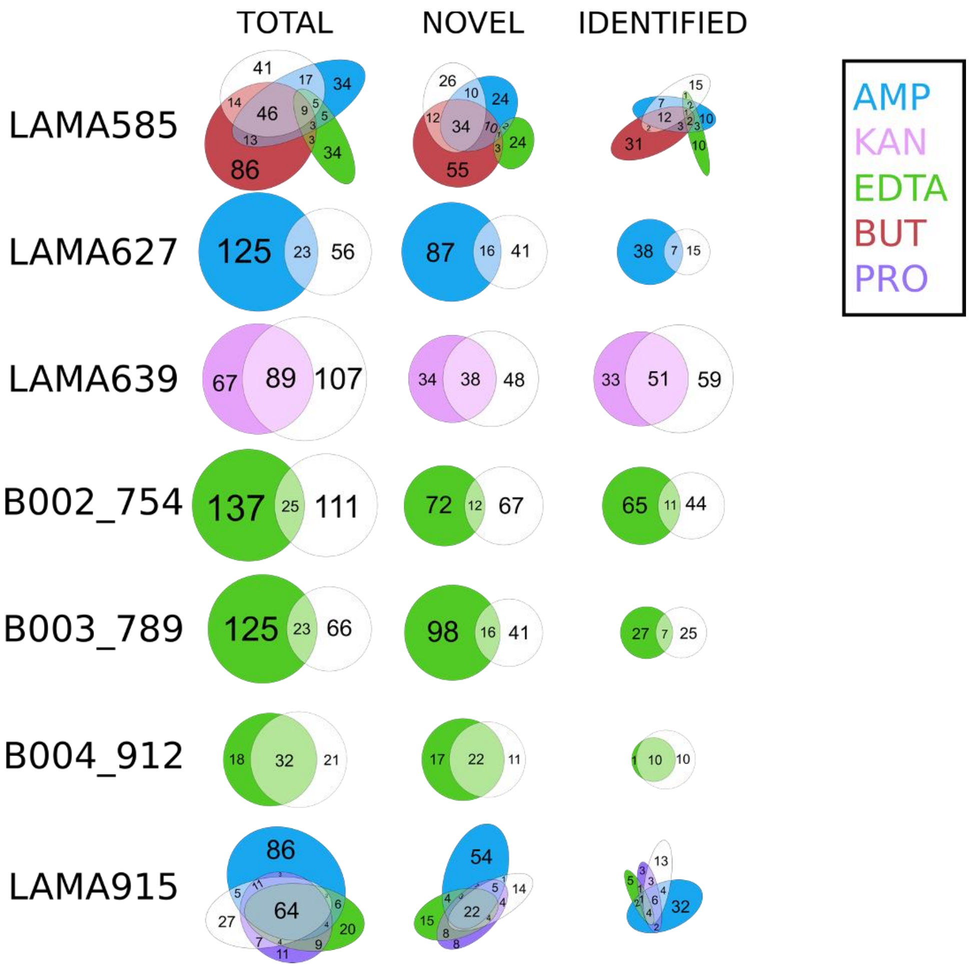

Figure S2. Euler diagrams of the 7 bacterial strains that compose the EPfB chemical library, grouping the metabolites detected in each strain by the chemical elicitor used in the bacterial cultures.

AMP: ampicillin (blue), KAN: kanamycin (pink), EDTA: ethylenediaminetetraacetic acid (green), BUT: sodium butyrate (red), PRO: procaine (purple). The control groups are shown in white. Each set is scaled according to the maximum number of metabolites in the dataset. Therefore, the size of the sets can be visually compared. The columns "total", "novel" and "identified" refer to the total number of different metabolites found in each strain (total), the number of identified (identified) and non-identified (novel) metabolites in MS/MS spectra databases consulted.

By observing the effect of a given elicitor to the different strains (**Figure S2**), it was possible to see that the same chemical elicitor displayed different efficiencies in inducing metabolites in the distinct bacterial strains. Ampicillin, for example, induced a large number of metabolites in LAMA915 and LAMA627, however its impact on LAMA585 metabolite production was less evident. LAMA 585 and LAMA915 were cultivated in the presence of different chemical elicitors. For LAMA915, ampicillin was the chemical elicitor that most induced metabolites in this strain. Whereas was less efficient in inducing metabolites in LAMA585, compared to the other elicitors used in the latter strain. About 62% and 19% of the elicited metabolites in LAMA915 and LAMA585, respectively, were exclusively detected in the ampicillin growth condition (**Table S3** and **Figure S3a**).

Table S3. Distribution of metabolites detected exclusively in the elicited conditions (upregulated) of the 7 bacterial strains used for the preparation of the EPfB library.

The number of potentially novel metabolites (not identified against MS/MS spectra databases) found in each strain is denoted in parenthesis.

Strain	Elicited Exclusive	AMP100 Exclusive	KAN100 Exclusive	EDTA10 Exclusive	PRO100 Exclusive	BUT50 Exclusive
LAMA585	178 (119)	34 (24)		34 (24)		86 (55)
LAMA627	125 (87)	125 (87)				
LAMA639	67 (34)		67 (34)			
B002_754	137 (72)			137 (72)		
B003_789	125 (98)			125 (98)		
B004_912	18 (17)			18 (17)		
LAMA915	139 (93)	86 (54)		20 (15)	11 (8)	

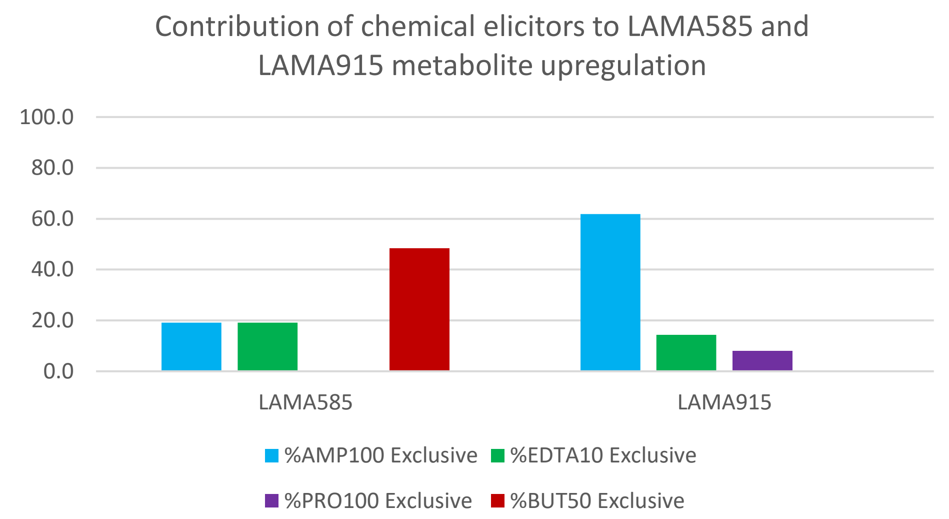

(a)

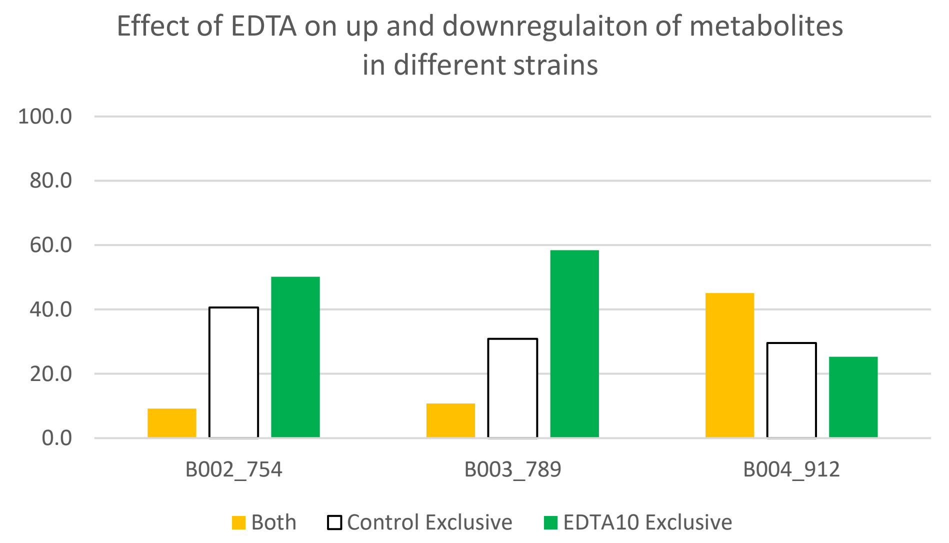

(b)

Figure S3. Distribution of elicited metabolites in different strains.

(a) Contribution of chemical elicitors to LAMA585 and LAMA915 metabolite upregulation, analyzed by the distribution of elicited exclusive metabolites in LAMA585 and LAMA915, per chemical elicitor used. AMP: ampicillin (blue), EDTA: ethylenediaminetetraacetic acid (green), BUT: sodium butyrate (red), PRO: procaine (purple). **(b)** Effect of EDTA on up and downregulaiton of metabolites in different strains, analyzed by the distribution of metabolites found exclusively in the control (downregulation) - white, or elicited (upregulation) – blue – growth conditions vs. both growth conditions (no modulation by the chemical elicitor) – yellow – of B002_754, B003_789 and B004_912.

The production of bacterial metabolites was also downregulated by the chemical elicitors in all strains, with some metabolites only observed in the control exclusive conditions (please refer to **Figure S2**, for an overview – the control groups in each Euler diagram are shown in white). On average 27.5% of the metabolites produced by each strain were downregulated by chemical elicitors, with a maximum of 40.7% of LAMA639 metabolites in the kanamycin condition *vs.* control, and 40.7% of B002_L754 metabolites in the EDTA condition *vs.* control –**Table S2**). LAMA585 and LAMA915, which were grown in more than one elicited condition, presented only about 10% of their metabolites repressed by chemical elicitors. Perhaps, the use of different chemical elicitors influenced this observation, highlighting that downregulation of bacterial metabolites can be elicitor dependent.

It was also possible to observe that the same chemical elicitor displayed differential modulation of the strain's metabolomes. For example, the strains B002_754, B003_789 and B004_912, in which EDTA was the only elicitor used, B004_912 was poorly influenced by EDTA; whereas B003_789 and B002_754 were highly regulated by this chemical elicitor. The latter two strains showed up or down regulation of ~90% their metabolites (in green and white respectively, **Figure S3b**), with only ~10% observed in both control and elicited conditions of the strain (yellow bars, **Figure S3b**). For B003_789 the use of EDTA on its cultures evidenced a large number of metabolites, with 58.4% being upregulated and only ~30% being repressed by EDTA. B002_754, displayed equilibrated up and downregulation by EDTA, with 50.2% of its metabolites induced and 40.7% repressed by this chemical elicitor. The effect of EDTA to B004_912 was less pronounced, with 45.1% of its metabolites detected independently of EDTA (detected in both growth conditions – in yellow, **Figure S3b**), only 25.4% upregulated (green bar), and about 30% downregulated (white bar) by this chemical elicitor.

It is worth noticing however that chemical elicitation modulated most of bacterial metabolites in all strains. On average, only 25.3% of the metabolites were observed in both control and elicited conditions, being thus not influenced by chemical elicitation.

In conclusion, the metabolism of the different strains used in the present work was modulated by chemical elicitors. However, the effect of a given chemical elicitor is strain dependent, and the number of metabolites repressed or induced by the chemical elicitor can vary according to the bacterial strain. In the same direction, a given strain can respond differently to distinct chemical elicitors, with a particular chemical elicitor working better (in terms of introducing different compounds to the cultures) to a given strain. Therefore, testing and selecting more than one chemical elicitor for a given bacterial strain growth under laboratory conditions is important towards the production of the maximum number of metabolites possible for a particular strain.

Novel compounds introduced by each strain

We next analyzed the percent of novel compounds introduced by each bacterial strain and by growth condition. On average, 62.7% of the metabolites produced *per* strain were novel (**Table S4** and **Figure S4**), with maximum of 72.4% (B003_789) and minimum of 45.6% (LAMA 639). The metabolites that were upregulated by chemical elicitors (found exclusively in the elicited conditions) showed the highest percent of novel metabolites, with 68.5% on average per strain, with maximum of 94.% (B004_912) and minimum of 50.7% (LAMA639). Importantly, at least half of the elicited metabolites were novel in all strains. Therefore, the elicited conditions showed advantages in inducing the production of novel over known (identified) metabolites in the elicited conditions over the control conditions [downregulated (exclusive to control conditions= 58.3%) and unmodulated (found in both control and elicited conditions= 59.2%)] - **Table S4**.

Table S4. Distribution of novel metabolites produced by each bacterial strain and the influence of chemical elicitation.

The numbers used in the calculations were derived from Table S1. Each cell in the table is colored according to its rank relative to the maximum value observed in each column (green scale: highest value in the column in green, lowest value in the column in white).

Strain	% novel/total per strain[a]	% novel/both per strain[b]	% novel/control per strain[c]	% novel/elicited per strain[d]
**LAMA585**	68.7	74.2	63.4	66.9
**LAMA627**	70.6	69.6	73.2	69.6
**LAMA639**	45.6	42.7	44.9	50.7
**B002_754**	55.3	48.0	60.4	52.6
**B003_789**	72.4	69.6	62.1	78.4
**B004_912**	70.4	68.8	52.4	94.4
**LAMA915**	56.2	41.5	51.9	66.9
average	***62.7***	***59.2***	***58.3***	***68.5***

[a] number of novel metabolites detected in a given strain, divided by the number of total metabolites in this strain.
[b] (not modulated by chemical elicitors): number of novel metabolites detected in both control and elicited growth conditions in a given strain, divided by the total number of metabolites detected in both control and elicited growth conditions of this strain.
[c] (downregulated by chemical elicitors): number of novel metabolites detected only in the control condition of a given strain, divided by the total number of metabolites detected only in the control growth condition of this strain.
[d] (upregulated by chemical elicitors): number of novel metabolites detected only in the elicited condition of a given strain, divided by the total number of metabolites detected only in the elicited growth condition of this strain.

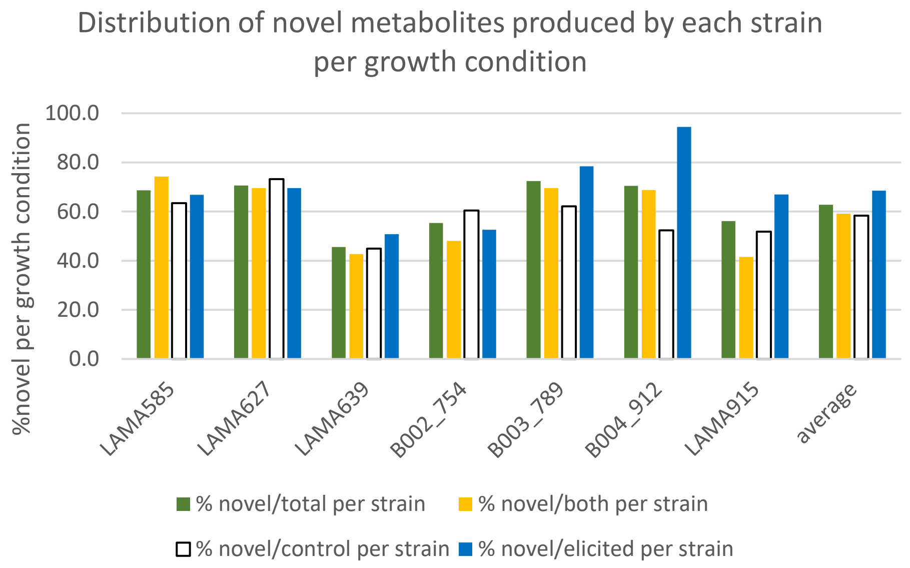

Figure S4. Distribution of novel metabolites detected in each strain.

Distribution of novel metabolites introduced per strain, according to the identification of the [M+H]+ spectra against databases. Percentages of non-identified compounds (novel) are shown in the different growth conditions: common to both control and elicited (yellow), detected exclusively in the elicited growth conditions (blue, upregulated) or exclusively in the control growth condition (white, downregulated). Data are plotted according to Table S4.

In conclusion, the novel metabolites were well distributed across the growth conditions used for preparing the EPfB library, with some advantage of the chemical elicitors in stimulating the production of novel metabolites by the different strains.

Metabolic exclusivity and redundancy among the bacterial strains

There was overlap (redundancy) of the metabolites detected in the different strains (please see **Figure S5** for an overview). From the 1,137 unique metabolites detected in the EPfB library, 807 (71% of the EPfB library) are exclusive to one specific bacterial strain (**Table S5**). We did not find any metabolite common to all bacteria. Less than 10% of the total metabolites detected were common to 3 or more bacteria and less than 20% were common to two bacteria (**Table S6**, and **Figure S6)**. This reflects that each individual strain brought different metabolites to the collection, expanding the chemical diversity, with about 30% of overall redundancy.

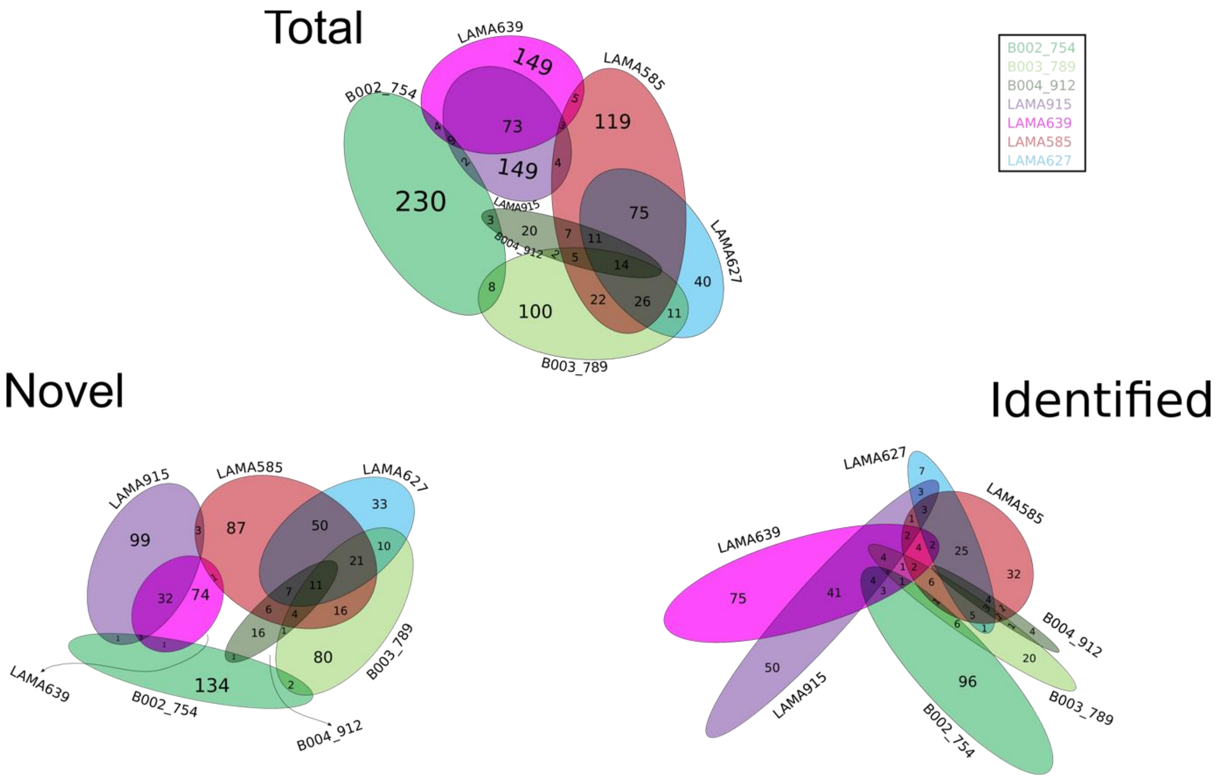

Figure S5. Euler diagrams of the metabolites produced by the different strains used for the construction of the EPfB library.

Three diagrams were constructed: "total", "novel" and "identified". The first is composed by the 1,137 metabolites detected in the EPfB library; "identified" refer to the 424 metabolites detected by LC-MS/MS, and identified in databases; whereas "novel" refer to the remaining 713 MS/MS spectra not identified in the databases. Some of the overlaps were excluded for clarity, please see tables and graphs for a precise quantitative analysis.

Table S5. Distribution of exclusive metabolites *per* strain in comparison to the whole EPfB library.

Exclusive metabolites are referred to the metabolites detected exclusively from samples derived from a given bacterial strain.

EXCLUSIVE TO	Total strain	% total EPfB library	Novel strain	% novel EPfB library	Identified strain	% identified EPfB library
LAMA585	119	10.5	87	12.2	32	7.5
LAMA627	40	3.5	33	4.6	7	1.7
LAMA639	149	13.1	74	10.4	75	17.7

B002_754	230	20.2	134	18.8	96	22.6
B003_789	100	8.8	80	11.2	20	4.7
B004_912	20	1.8	16	2.2	4	0.9
LAMA915	149	13.1	99	13.9	50	11.8
TOTAL	***807***	***71.0***	***523***	***73.4***	***284***	***67.0***

[a] relative to the total number of metabolites in the EPfB library (n=1,137)
[b] relative to the number of novel metabolites in the EPfB library (n=713)
[c] relative to the number of idnetified metabolites in the EPfB library (n=424)

Table S6. Distribution of redundant metabolites across the 7 bacterial strains used for the preparation of the EPfB library.

Redundant metabolites are referred to the metabolites detected from samples derived from two or more bacterial strains.

FOUND IN:	Total	Total (%)[a]	Novel	Novel (%)[b]	Identified	Identified (%)[c]
6 strains	1	0.1	0	0.0	1	0.2
5 strains	3	0.3	2	0.3	1	0.2
4 strains	21	1.8	14	2.0	7	1.7
3 strains	79	6.9	46	6.5	33	7.8
2 strains	226	19.9	128	18.0	98	23.1
TOTAL	***330***	***29.0***	***190***	***26.6***	***140***	***33.0***

[a] relative to the total number of metabolites in the EPfB library (n=1,137)
[b] relative to the number of novel metabolites in the EPfB library (n=713)
[c] relative to the number of idnetified metabolites in the EPfB library (n=424)

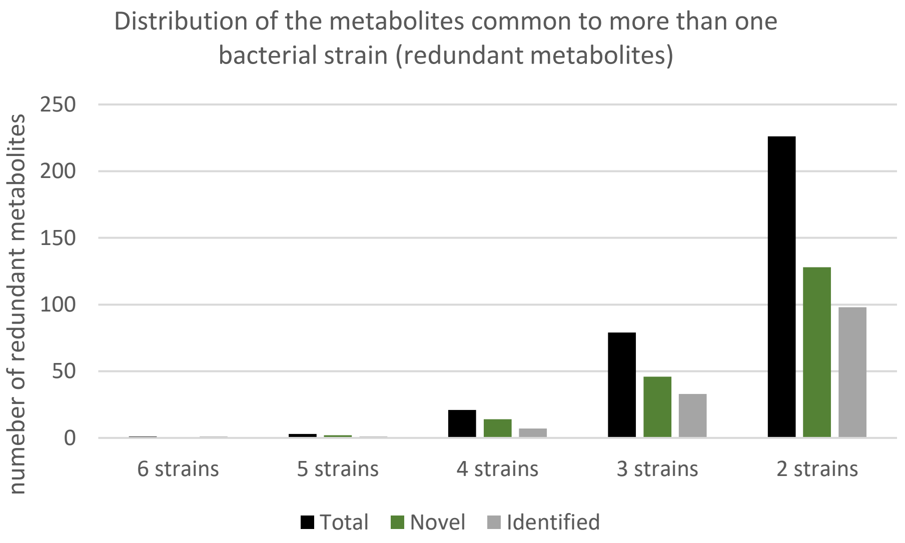

(a)

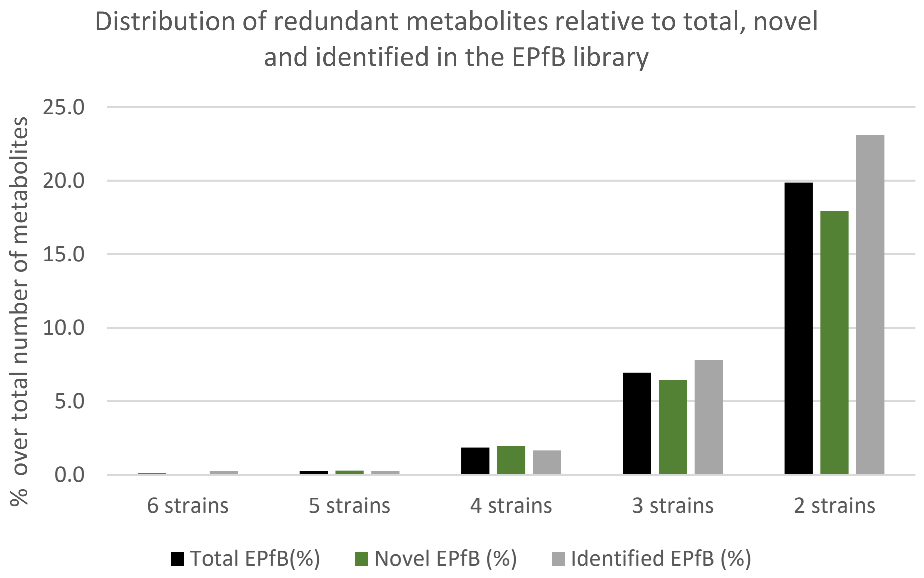

(b)

Figure S6. Metabolite redundancy among the 7 strains that compose the pilot EPfB library.

Data are plotted according to Table S6. **(a)** Absolute values. **(b)** Percentage relative to the total number of metabolites detected in the EPfB library (n=1,137 unique metabolites, n=713 novel metabolites and n=424 identified metabolites).

It is worth noticing that LAMA915, LAMA639 and B002_754 were the strains that showed the least overlaps library (**Figure S5** – "total", **Table S7**), reflecting a higher contribution of these strains to the whole EPfB (**Table S5**). Interestingly, these three strains are the strains that showed biological activity in our biological screens (Table 1 main paper).

B002_754 bring a large number of exclusive (n=230) and novel (n=134) metabolites (**Table S7**), with low overlap with the other strains (**Figure S5**). LAMA915 and LAMA639 further introduced compounds to the EPfB library (n=149 each, **Table S7**), partially overlapping with each other in the context of novel compounds (**Figure S5** – "novel"); still, 38.1% and 28.1% of the metabolites exclusively detected in LAMA915 and LAMA639 samples were novel in comparison to databases (**Table S7**).

On the other hand, LAMA627 was the strain that showed the highest redundancy, with less than 20% of its metabolites being exclusive to this strain. Furthermore, only 16.2% of the metabolites exclusively produced by LAMA627 were novel in comparison to databases (**Table S7**), reflecting a less interesting strain in the collection.

Table S7. Distribution of exclusive and novel metabolites across the 7 bacterial strains used for the preparation of the EPfB library.

Exclusive metabolites are referred to the metabolites detected exclusively from samples derived from a given bacterial strain. Each cell in the table is colored according to its rank relative to the maximum value observed in each column (green scale: highest value in the column in green, lowest value in the column in white).

EXCLUSIVE TO:	Total strain	Exclusive strain	% exclusive strain	Novel strain	% novel strain
**LAMA585**	316	119	37.7	87	27.5
**LAMA627**	204	40	19.6	33	16.2
**LAMA639**	263	149	56.7	74	28.1
**B002_754**	273	230	84.2	134	49.1
**B003_789**	214	100	46.7	80	37.4

**B004_912**	71	20	28.2	16	22.5
**LAMA915**	260	149	57.3	99	38.1

Novelty of the strain exclusive metabolites

About 65% of the strain exclusive metabolites are novel (523/807), which corresponds to 73.4% of the whole EPfB library novelty (**Table S5** and **Figure S7** – "total group"), showing the novelty of the library is mostly influenced by the strain exclusive metabolites.

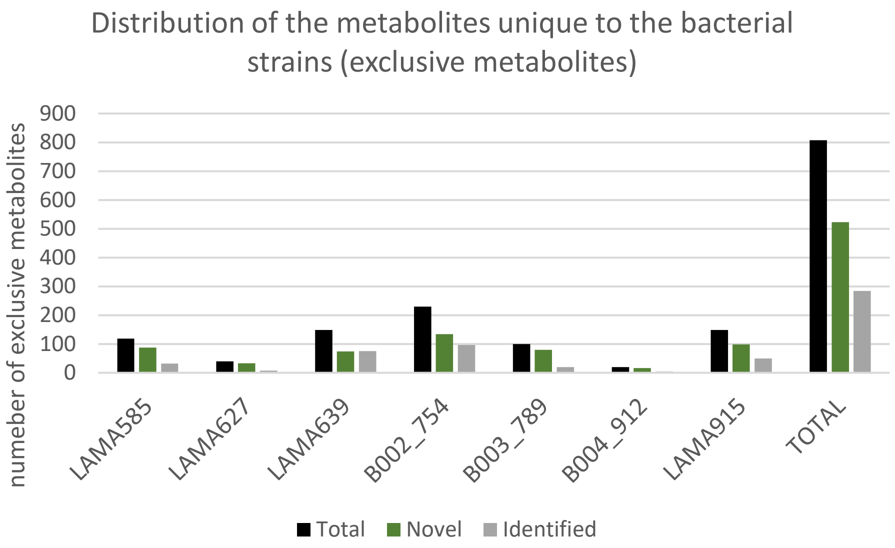

(a)

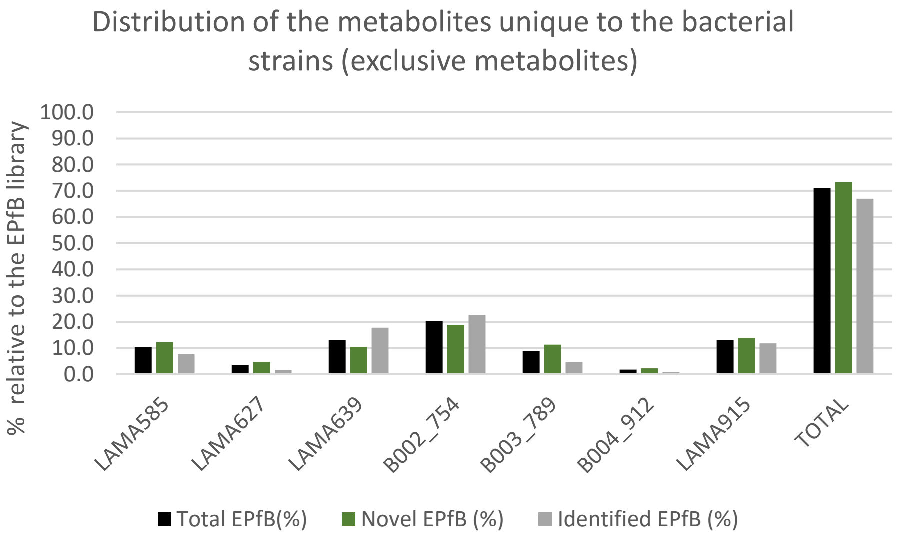

(b)

Figure S7. Metabolites that were exclusively detected in one of the 7 strains that compose the pilot EPfB library.

Data are plotted according to Table S5. **(a)** Absolute values. **(b)** Percentage relative to the total number of metabolites detected in the EPfB library (n=1,137 unique metabolites, n=713 novel metabolites and n=424 identified metabolites).

The strain B002_754 was the one that contributed the most to the unique metabolites found in the EPfB library (**Figure S7**). B002_754 produced 273 metaboites, 230 being exclusive to this strain (84.2% -**Table S7**). The 230 metabolites introduced by B002_754, correspond to 20.2% of the total metabolites of the EPfB library (230/1,137) – **Table S5** and **Figure S7b**. From the metabolites introduced by this strain, more than a half were not identified with the databases used (134/230= 58.3% of potentially novel metabolites), corresponding to 18.8% of the novelty of the EPfB library. LAMA585, on the other hand, the bacteria that produced the highest number of metabolites in the EPfB library (336, 27.8% of the whole EPfB library) showed only 119 (37.7%) exclusive metabolites, the other 217 metabolites being redundant to other strains. With this, LAMA585 contributed only with 10.5% of the total metabolites in the EPfB library (119/1,137). However, 73% (87/119) of these LAMA585 exclusive metabolites are potentially novel bacterial compounds, highlighting LAMA585 as one of the bacterial strains that most contributed to the novelty of the library (12.2%). In this context, LAMA915 is another bacterial strain that must be discussed. It introduced 149 metabolites to the EPfB library (72.3% of its metabolites and 13.1% of the total metabolites of the library) with about 66% of these (99/149) being novel compounds, thus contributing to 13.9% of the EPfB library's novelty. LAMA639 contributed to the same number of exclusive metabolites as LAMA915 (149 compounds), however, with less novelty to the EPfB library (10.4%), as half of its exclusive metabolites were identified in databases.

Regarding the metabolites that were found redundant to more than one strain, a little more than a half (57.6%) were not identified in the databases used (190/330 potentially novel metabolites that are redundant to more than one bacterial strain) - **Table S6**. Overall, the distribution of novel *vs.* identified redundant metabolites was similar within the different combinations analyzed (6-2 strains), with a bit more than a half of the redundant metabolites per combination being novel (**Figure S6** and **Table S6a**). The redundant metabolites correspond to 26.6% of the novel (190/713) and 33.0% of the identified (140/424) metabolites detected in the EPfB library (**Figure S6**). The different combinations used for the analyses of redundant metabolites, in 2-6 strains, show that non identified metabolites are mostly present over novel metabolites, in the combination of 2 strains – **Figure S6b**. The complete list of metabolites per bacterial strain and combination of common metabolites to more than one strain is presented in **Table S8**.

Table S8. Common metabolites for different strains.

strain or combination of strains	Total	Novel	Identified
LAMA585 & LAMA627 & B002_754 & B003_789 & B004_912 & LAMA915	1	0	1
LAMA585 & LAMA627 & LAMA639 & B003_789 & LAMA915	1	1	0
LAMA585 & LAMA627 & B002_754 & B003_789 & LAMA915	1	1	0
LAMA585 & LAMA639 & B002_754 & B003_789 & LAMA915	1	0	1
LAMA585 & LAMA627 & LAMA639 & B003_789	1	1	0
LAMA585 & LAMA627 & B002_754 & B003_789	1	0	1
LAMA585 & LAMA627 & B002_754 & B004_912	2	1	1
LAMA585 & LAMA627 & B003_789 & B004_912	14	11	3
LAMA585 & B002_754 & B003_789 & B004_912	1	1	0
LAMA585 & B002_754 & B004_912 & LAMA915	1	0	1
LAMA639 & B003_789 & B004_912 & LAMA915	1	0	1
LAMA585 & LAMA627 & LAMA639	2	0	2
LAMA585 & LAMA627 & B002_754	2	1	1
LAMA585 & LAMA627 & B003_789	26	21	5
LAMA585 & LAMA639 & B003_789	3	1	2
LAMA627 & LAMA639 & B003_789	3	3	0
LAMA585 & B002_754 & B003_789	2	1	1
LAMA639 & B002_754 & B003_789	1	0	1
LAMA585 & LAMA627 & B004_912	11	7	4
LAMA585 & B003_789 & B004_912	5	4	1
LAMA627 & B003_789 & B004_912	2	1	1
LAMA585 & LAMA627 & LAMA915	4	1	3
LAMA585 & LAMA639 & LAMA915	3	1	2
LAMA639 & B002_754 & LAMA915	9	5	4
LAMA639 & B003_789 & LAMA915	4	0	4
B002_754 & B003_789 & LAMA915	2	0	2
LAMA585 & LAMA627	75	50	25
LAMA585 & LAMA639	5	1	4

**LAMA627 & LAMA639**	2	0	2
**LAMA585 & B002_754**	2	1	1
**LAMA639 & B002_754**	4	1	3
**LAMA585 & B003_789**	22	16	6
**LAMA627 & B003_789**	11	10	1
**LAMA639 & B003_789**	1	0	1
**B002_754 & B003_789**	8	2	6
**LAMA585 & B004_912**	7	6	1
**LAMA627 & B004_912**	1	1	0
**B002_754 & B004_912**	3	1	2
**B003_789 & B004_912**	2	1	1
**LAMA585 & LAMA915**	4	3	1
**LAMA627 & LAMA915**	4	1	3
**LAMA639 & LAMA915**	73	32	41
**B002_754 & LAMA915**	2	2	0
**LAMA585**	119	87	32
**LAMA627**	40	33	7
**LAMA639**	149	74	75
**B002_754**	230	134	96
**B003_789**	100	80	20
**B004_912**	20	16	4
**LAMA915**	149	99	50
TOTAL	***1137***	***713***	***424***

In conclusion, the metabolites that were exclusive to a given strain brought, in general, more novelty to the EPfB library (523 novel metabolites that where exclusive to a given strain *vs.* 190 novel common to more than one strain). The strains B002_754, LAMA915 and LAMA585 brought the higher percentages of strain exclusive and novel compounds (18.8%, 13.9% and 12.2% of the EPfB library's novelty, respectively).

Chemical diversity introduced by each bacterial strain

We next analyzed the chemical diversity introduced by each strain to the EPfB library using spectra similarity molecular networks (SSMN). The contribution of the chemical elicitors to each strain is also contemplated in this analysis (**Figure S8**).

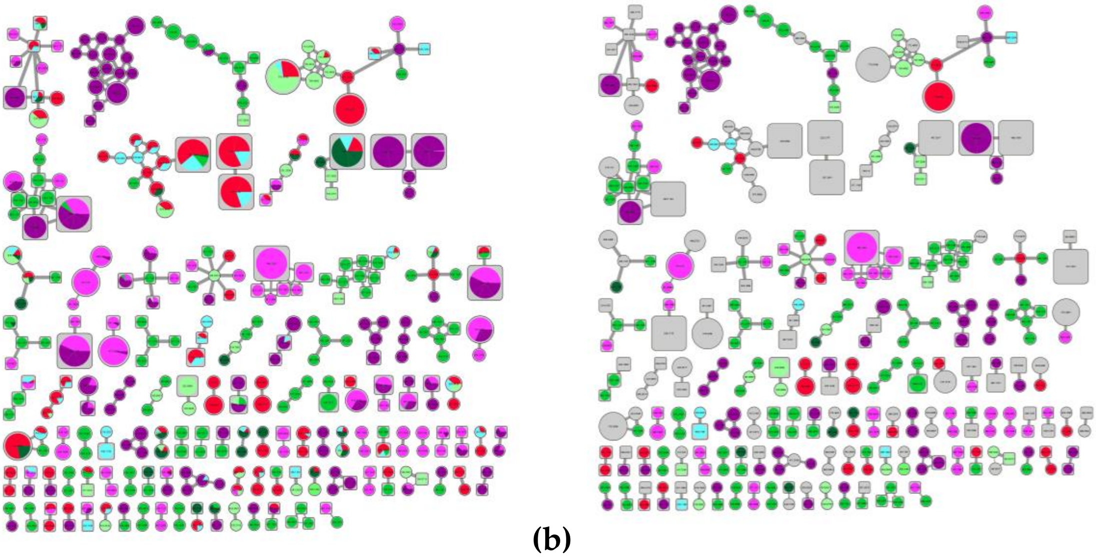

(a) **(b)**

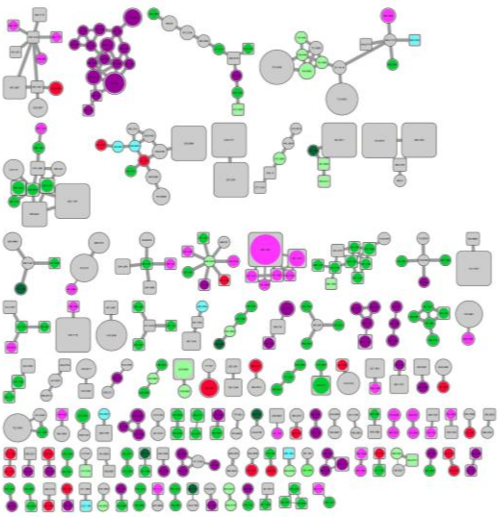

(c)

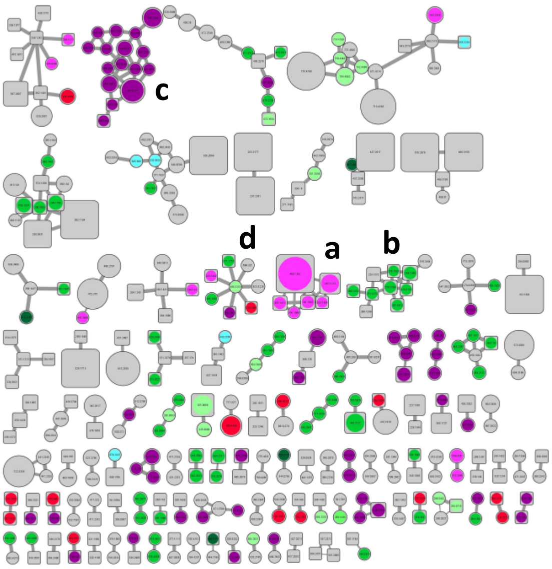

(d)

Figure S8. SSMN of the EPfB library colored by the producing bacteria.

Metabolites derived from the different strains are colored as follows: *B002_754*, *B003_789*, *B004_912*, *LAMA915*, *LAMA639*, *LAMA585*, *LAMA627*. **(a)** Whole network showing redundant and exclusive metabolites produced by the different bacterial strains. A pie chart is shown to highlight nodes originated to more than one bacteria. **(b)** SSMN highlighting the nodes detected exclusively in a given bacteria. The nodes of redundant metabolites (found in samples from more than one bacterial strain) are colored grey. **(c)** SSMN highlighting the metabolites modulated by chemical elicitors. The nodes detected in both control and elicited samples are also colored grey. **(d)** SSMN highlighting the metabolites exclusively produced by the different bacterial strains and that were upregulated by chemical elicitors (found exclusively in the elicited conditions). The nodes detected in the control exclusive samples (downregulated by the chemical elicitors used) are also colored grey.

LAMA915, LAMA639 and B002_754 showed clusters of metabolites that were exclusive to these strains (**Figure S8b**, in purple, pink and green, respectively). It is thus clear the contribution of these strains to the chemical diversity of the EPfB library. Some of these clusters were modulated by chemical elicitors (**Figure S8c**), being upregulated by chemical elicitors (exclusively found in the elicitation conditions - **Figure S8d**). These clusters represent: “a” JBIR35 and tryptoquivaline K discussed in the main paper, “b” phenylalanine derivatives and “c” possible biotransformation products of ampicillin. Cluster “d” is also highlighted as induced by chemical elicitation and presenting compounds produced by different bacteria. Cluster “d” is represented by diketopiperazines. Four other small clusters can also be highlighted with the characteristics of cluster “d” – **Figure S9**. Additionally, an analysis of the strain exclusive nodes revealed that only two metabolites were elicited by the same bacteria with more than one chemical elicitor (**Figure S9**, pointed by the arrows).

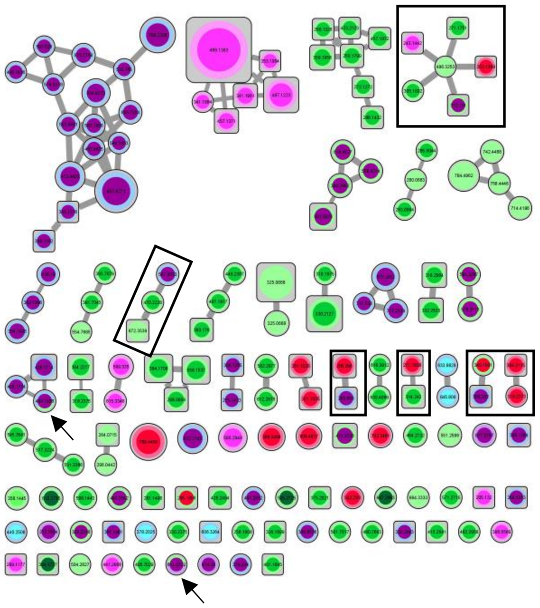

Figure S9. SSMN of the metabolites that were strain exclusive and upregulated by chemical elicitors. Each node is colored according to the bacterial strain it was derived (***B002_754***, *B003_789*, ***B004_912***, ***LAMA915***, *LAMA639*, ***LAMA585***, *LAMA627*) and surrounded by colors relative to the chemical elicitors used. AMP: light blue, KAN: light pink, EDTA: light green, PRO: violet, BUT: red. If more than one color circulating the node appears it means it was induced by more than one chemical elicitor. Two nodes rely on this case and are pointed by the arrows. Clusters composed by nodes formed by metabolites produced by different bacteria are boxed (6 clusters).

Chemical space coverage of the strain exclusive upregulated metabolites

We further analyzed the contribution of the strain exclusive upregulated metabolites to the chemical space of the EPFB library. For this, the PCA plot of the EPfB library prepared with the UNPD-ISDB and DrugBank molecules was used for reference (**Figure S10**).

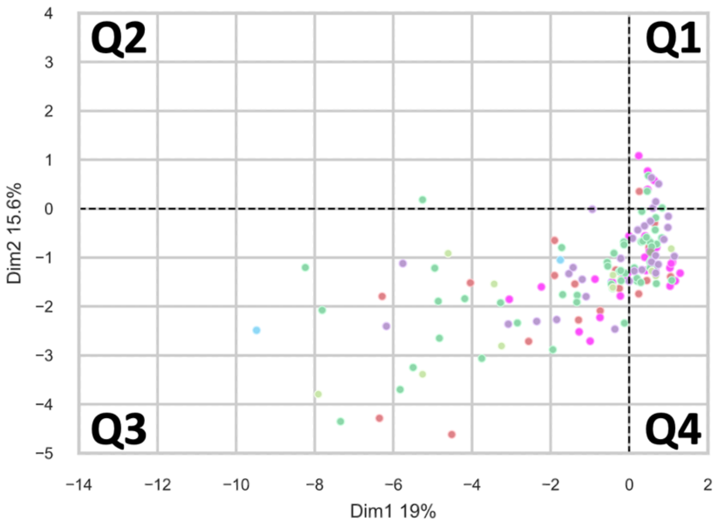

(a)

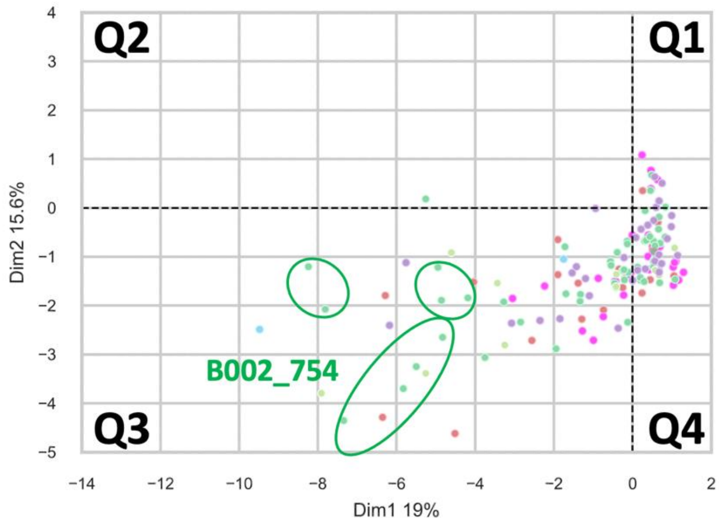

(b)

Figure S10. Chemical space of the upregulated and strain exclusive bacterial metabolites in the EPfB library.

The PCA are the same as shown in Figure 6 of the main paper, however highlighting only the "strain exclusive upregulated" metabolites. **(a)** Strain exclusive upregulated compounds that were identified in databases are shown in dots and colored according to the strain of origin: ***B002_754***, *B003_789*, ***B004_912***, ***LAMA915***, ***LAMA639***, ***LAMA585***, *LAMA627*. **(b)** Same as "a", highlighting compounds from B002_754 exclusively detected in the EDTA growth condition.

It is possible to observe upregulated metabolites spread across the chemical space of the EPfB library, specially at the regions relevant for approved drugs (Q3 and Q4) - **Figure S10**. It is also important to comment that some strains concentrated upregulated metabolites In specific regions of the chemical space. The upregulated metabolites of B002_754, for example, covered Q3 with 3 regions being highlighted in **Figure S10b**. These represent nitrogenated compounds identified as nucleoside derivatives, cyclic peptides and linear guanidines.

These compounds are not clustered in the SSMN, appearing as self-loops (not shown in **Figure S8** and **Figure S9**). As comment in the main paper, the evaluation of the chemical space coverage of identified compounds in a given collection is very valuable to sense the chemical features and chemical diversity of the collection. The comparative analyses of the chemical space of the EPfB library, and its subsets, with the approved drugs (DrugBank) and UNPD chemical spaces used in the present work evidenced the biological relevance of the collection. These analyses complement the SSMN analyses, highlighting chemical features of the collection that would be missed by SSMN analyses alone. The example presented in **Figure S10** clearly illustrate this, with the nitrogenated compounds produced by B002_754 exclusively in the presence of EDTA being evidenced by chemical space coverage analysis in Q3 - a chemical space region very relevant for approved drugs.

General conclusion of the strain specific analyses

In conclusion, the analyses of the EPfB library *per* strain show that the metabolism of the different strains used in the present work was modulated by chemical elicitors. However, the effect of a given chemical elicitor was strain dependent, and the number of metabolites induced or repressed by the chemical elicitor varyed according to the bacterial strain. In the same direction, a given strain responded differently to distinct chemical elicitors, with a particular chemical elicitor working better to one or other strain. Therefore, testing and selecting more than one chemical elicitor for a given bacterial strain growth under laboratory conditions is important towards the production of the maximum number of metabolites possible for a particular strain.

It is also important to comment that novel metabolites were well distributed across the growth conditions used for preparing the EPfB library, with some advantage to chemical elicitors in introducing novel metabolites to the collection.

The strains B002_754, LAMA915 and LAMA639, that showed samples displaying biological activity in our screens, were the strains that most contributed to the EPfB library. These strains produced the highest number of unique metabolites in the EPfB library. The metabolites that were exclusive to a given strain brought, in general, more novelty to the EPfB library, with B002_754, LAMA915 and LAMA585 bringing the higher percentages of strain exclusive and novel compounds. Strains LAMA915, LAMA639 and B002_754 also introduced families of compounds to the EPfB library (exclusive clusters in the SSMN), expanding the chemical diversity of the collection. Some of these clusters were modulated by chemical elicitors, being upregulated by these chemicals. B002_754, in particular showed upregulation of nitrogenated compounds by EDTA, which are in the most relevant region of the approved drug's chemical space.
